# Intracellular Parasitic Infections Caused by *Plasmodium falciparum*, *Leishmania* spp., *Toxoplasma gondii*, *Echinococcus multilocularis*, Among Key Pathogens: Global Burden, Transmission Dynamics, and Vaccine Advances—A Narrative Review with Contextual Insights from Armenia

**DOI:** 10.3390/vaccines13111082

**Published:** 2025-10-22

**Authors:** Tatevik Sargsyan, Lala Stepanyan, Avetis Tsaturyan, Rosanna Palumbo, Caterina Vicidomini, Giovanni N. Roviello

**Affiliations:** 1 Scientific and Production Center “Armbiotechnology” NAS RA, 14 Gyurjyan Str., Yerevan 0056, Armenia; tatev-sargsyan@ysu.am (T.S.);; 2 Institute of Pharmacy, Yerevan State University, 1 Alex Manoogian Str., Yerevan 0025, Armenia; 3 Institute of Biostructures and Bioimaging, National Research Council (CNR), 80145 Naples, Italy

**Keywords:** transmission dynamics, One Health, public health challenges, Armenian epidemiology, host, obligate, facultative

## Abstract

Intracellular parasitic infections continue to pose significant public health and veterinary challenges globally, driven by their ability to evade immune responses, persist within host cells, and spread through complex transmission pathways. Caused by a diverse array of protozoan, helminthic, and arthropod-borne parasites, these infections, such as toxoplasmosis, leishmaniasis, and tick-borne diseases, remain prevalent across many regions, often exacerbated by environmental, socio-economic, and ecological factors. This review explores the current knowledge on intracellular parasitic diseases, outlining parasite classification, immune evasion mechanisms, diagnostic difficulties, and control strategies. Special attention is given to recent advancements in vaccine development, with a focus on experimental and licensed vaccines targeting intracellular pathogens. Additionally, the review highlights the importance of a ‘One Health’ approach, integrating human, animal, and environmental health efforts to address the multifaceted nature of parasitic transmission and control. Within this global context, Armenia serves as a case study, offering insight into how local ecological conditions, vector distribution, public health capacity, and social determinants shape the national burden of these infections. Challenges in Armenia, such as limited access to advanced diagnostics, underreporting, and the need for robust surveillance systems, underscore broader regional needs for investment in research, infrastructure, and cross-sectoral collaboration.

## 1. Introduction

Intracellular parasite infections are a big threat to global health because they can penetrate and live inside host cells without being recognized by the immune system. This makes diagnosis and treatment more difficult. Pathogens, such as protozoa like *Leishmania* species, *Toxoplasma gondii*, and *Plasmodium* species, as well as some helminths and arthropod-borne agents, make people sick, kill them, and cause long-term problems around the world [1,2,3,4]. Soil-transmitted helminths affect more than 1.5 billion people around the world. Toxoplasmosis alone causes more than 800,000 Disability-Adjusted Life Years (DALYs) each year. All of the main parasite diseases cause about 9 million Disability-Adjusted Life Years (DALYs). Intracellular parasites spread in many ways, such as through bites from vectors, eating or drinking contaminated food or water, and zoonotic cycles that connect people, animals, and wildlife. Poverty, poor sanitation, climate change, and environmental disruptions are just a few of the many social and ecological factors that have a big impact on how quickly and widely diseases spread [2,5,6,7,8]. These factors coming together lead to a high rate of co-infections. For example, helminths and protozoa can be found together in virus-infected populations (such as HIV and HBV) up to 30% of the time, which makes the disease worse and makes it harder to treat [9,10,11]. Intracellular pathogens have come up with special ways to avoid the immune system, like changing the way antigens are presented, changing the way the host’s immune system works, and changing the way apoptosis works. All of these make standard tests and treatments less effective [12,13]. Accurate detection continues to pose challenges, especially in resource-limited settings because of restricted serological cross-reactivity, the sensitivity of microscopy, and the limited availability of molecular tools. As a result, control strategies must be all-encompassing, including better diagnostics, health education, vector control, surveillance, mass drug administration, and environmental sanitation [14,15]. The advancement of innovative therapeutic strategies for addressing socially significant diseases increasingly focuses on molecular platforms, incorporating both natural and synthetic compounds, including peptides, oligonucleotides, and hybrid structures such as nucleopeptides, with a growing interest in their potential applications against parasitic infections [16,17,18,19,20,21,22,23,24,25]. The advancement of vaccines targeting intracellular parasites is underway, with numerous candidates presently under examination or approved (e.g., vaccines for *Toxoplasma*, *Leishmania*, and *Plasmodium*), supported by novel methodologies including live-attenuated strains, recombinant antigens, and viral vectors. These innovative platforms aim to establish enduring cell-mediated immunity, a crucial defense against infections within cells. The “One Health” framework, which links the health of people, animals, and the environment, is becoming more and more important for figuring out how zoonotic diseases spread and how to stop them for good [26,27,28]. In this worldwide setting, Armenia exemplifies a critical case study of regional epidemiology shaped by unique ecological and socio-economic factors. Visceral leishmaniasis has reemerged in Armenia since 1999, with 167 reported cases by 2019. Molecular studies have indicated that the cause is *Leishmania infantum*. Vector and reservoir mapping remains insufficient, despite initial entomological research identifying *Phlebotomus* spp. in prevalent regions [29,30,31,32]. Tick-borne pathogens such as *Babesia* and *Theileria* have been detected in livestock and companion animals, signifying significant zoonotic risks [33,34]. Parasitic infections remain a significant public health challenge in Armenia, aggravated by the relatively high prevalence of pathogens such as enterobiasis, ascariasis, and echinococcosis, alongside persistent underdiagnosis and delayed case detection. The limited availability of modern molecular diagnostics, insufficient laboratory capacity, and inadequate intersectoral coordination continue to impede effective surveillance and timely intervention, with both diagnostic capabilities and research in this domain remaining underdeveloped in the Caucasus region. Addressing these challenges requires a multifaceted approach that includes enhancing laboratory infrastructure, training healthcare professionals in modern diagnostic techniques, and fostering collaboration between public health and veterinary sectors. By prioritizing these efforts, Armenia can improve its capacity to detect and respond to parasitic infections, ultimately safeguarding public health and reducing the burden of zoonotic diseases. In addition, leveraging international partnerships and funding can accelerate the implementation of these strategies, ensuring that Armenia is equipped to tackle emerging health threats. Strengthening data collection and sharing practices will also play a crucial role in tracking disease patterns and facilitating informed decision-making. This review offers a comprehensive analysis of the present epidemiological landscape in Armenia, highlighting critical diagnostic and research deficiencies and investigating principal challenges and prospective strategies for enhancing control measures. The review examines the burden, immunopathogenesis, diagnostic deficiencies, and advancements in vaccination for intracellular parasitic diseases, utilizing both global and local data. It illustrates the importance of integrated “One Health” strategies for sustainable disease management in Armenia and comparable settings. These strategies not only promote collaboration among human, animal, and environmental health sectors but also facilitate the implementation of targeted interventions. By addressing the complex nature of these diseases, stakeholders can work towards more effective prevention and treatment protocols that ultimately improve public health outcomes [35,36,37].

### Methodology

This review was conducted through a comprehensive and systematic examination of scientific literature and official sources, integrating both global and regional perspectives. An extensive search was performed across major scientific databases, including Web of Science, PubMed, Scopus, and Google Scholar, covering publications from the early 20th century to September 2025. Historical epidemiological data, particularly on diseases such as malaria and leishmaniasis, were retrieved to contextualize long-term trends and developments. Primary emphasis was placed on literature published between 2010 and 2025, reflecting the most recent advancements and findings relevant to the scope of this review. Articles retrieved from these research engines were available in English, ensuring accessibility and consistency in scientific interpretation. In addition to internationally indexed sources, targeted efforts were made to incorporate region-specific publications from Russia, Armenia, and neighboring countries. These included peer-reviewed articles, government reports, epidemiological surveys, and conference proceedings, accessed both online and in print formats, in Armenian and Russian languages. To ensure a balanced representation of both global and local health landscapes, official data and publications from the World Health Organization (WHO) and the Ministry of Health of the Republic of Armenia were critically analyzed. These sources provided validated statistics and policy frameworks essential for understanding disease prevalence, public health responses, and regional challenges. We conducted our literature search using the keywords “intracellular parasitic infections,” “transmission dynamics,” “vaccine development,” “Armenia,” and pathogen-specific terms such as *Plasmodium falciparum*, *Leishmania* spp., *Toxoplasma gondii*, and *Echinococcus multilocularis*. As mentioned above, the search covered publications from the early 20th century to September 2025, with a primary focus on articles published between 2010 and 2025, while the inclusion criteria were: relevance to intracellular parasitic infections, availability in English (for indexed databases), or in Armenian/Russian (for regional sources), and publication in peer-reviewed journals or official government outlets. Exclusion criteria included retracted papers, non-scientific commentary, and duplicate records. Evidence appraisal was based on source credibility, epidemiological relevance, and contribution to understanding transmission or vaccine strategies.

## 2. Global Overview of Intracellular Parasitic Infections: Classification, Transmission and Immune Evasion

### 2.1. Classification of Intracellular Parasitic Infections

Intracellular parasites are chiefly categorized according to their obligatory or facultative intracellular characteristics, host specificity, and phylogenetic classification. These categories are essential for comprehending parasite biology, pathogenicity, and targeted therapy. Recent literature emphasizes molecular adaptations and immune evasion strategies that further refine these classifications [38,39,40,41,42,43]. Parasites are categorized into two primary groups based on intracellular dependency. Obligate intracellular parasites necessitate host cells for their survival and replication. Some examples are *Plasmodium* spp., *Toxoplasma gondii*, and *Cryptosporidium* spp., which need the inside of cells to grow and avoid the immune system [44,45,46]. Facultative intracellular parasites can endure both within and outside of host cells. *Leishmania* spp. and *Trypanosoma cruzi* are representatives of this group, demonstrating both intracellular and extracellular life cycle stages [41,47,48,49]. Parasites are also grouped by their taxonomic lineage. In this regard, intracellular parasites predominantly fall into the following groups:

(i) Protozoa (Eukaryotes);
(ii) Apicomplexa: *Plasmodium*, *Toxoplasma*, *Cryptosporidium;*
(iii) Kinetoplastida: *Leishmania*, *Trypanosoma;*
(iv) Microsporidia: Spore-forming unicellular parasites (e.g., *Enterocytozoon bieneusi*);
(v) intracellular bacteria: e.g., Chlamydia, *Rickettsia*, and *Coxiella* [41,50,51,52].

Parasites are also different from each other in the types of hosts they can live in. Some parasites only infect certain hosts (for example, *T. gondii* only reproduces in cats), while others, like *Plasmodium falciparum*, only infect humans. Zoonotic parasites infect both animals and humans, making it harder to get rid of them [50,53,54].

To provide a comparative overview, Table 1 summarizes representative intracellular parasites, their phylogenetic classification, intracellular dependency, definitive and intermediate hosts, and primary modes of transmission.

**Scheme 1:**
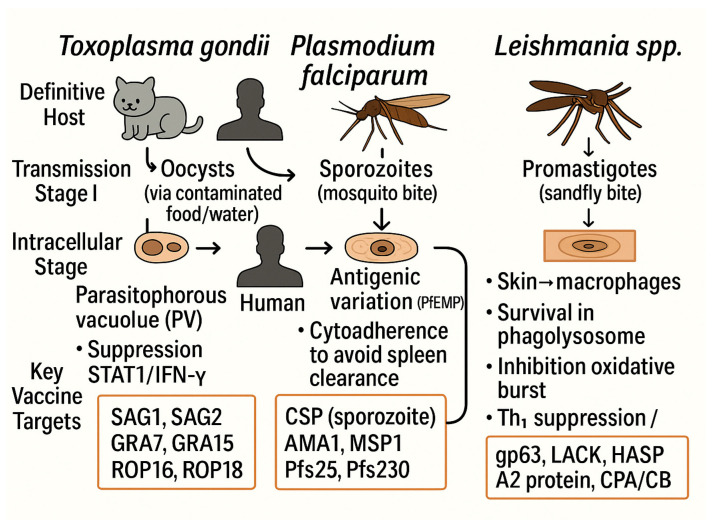
Schematic representation of the parasite life cycle and main vaccine strategies for the following targets: *Toxoplasma*, *Plasmodium*, and *Leishmania*.

As shown in Table 1, these parasites exhibit distinct host specificities and transmission strategies, but they can also be grouped according to their life cycle complexity, as described below. The scheme below (Scheme 1) shows the life cycle stages, immune evasion mechanisms, and vaccine targets of the most important protozoan parasites.

The examples above show that each parasite has a specific host and method of spreading, but they can be grouped by life cycle complexity. The number of hosts needed for their growth and spread makes this complex.

(i) Direct (monoxenous) (single host): Parasites live their whole lives in one host organism without needing an intermediate host. Some examples are *Cryptosporidium* spp., *Giardia lamblia*, and *Entamoeba histolytica*.
(ii) Indirect (heteroxenous) (Multi-host): Parasites need more than one host to finish their life cycle. *Plasmodium* spp. (human + mosquito), *Leishmania* spp. (human/animal + sandfly), *Toxoplasma* gondii (cat + intermediate hosts), and *Echinococcus multilocularis* (dog/fox + rodent/human) are some examples [55,56].

**Table 1:** Key characteristics of selected parasites, including type, host specificity, and transmission routes.

Parasite	Type	Obligate/ Facultative	Definitive Host(s)	Intermediate Host(s)	Transmission/Vector	Ref.
*Plasmodium falciparum*	Protozoa (Apicomplexa)	Obligate	*Anopheles* mosquitoes (the sexual cycle takes place in them)	Humans (hepatocytes, RBCs)	Mosquito bite (sporozoite)	[52,54]
*Toxoplasma gondii*	Protozoa (Apicomplexa)	Obligate	Felidae family (e.g., domestic and wild cats)	Humans, many warm-blooded animals	Oral (oocysts, tissue cysts)	[46,53]
*Leishmania donovani*	Protozoa (Kinetoplastida)	Obligate/ Facultative *	Humans	Sand flies (*Phlebotomus*, *Lutzomyia*); reservoirs: canines, rodents, mammals	Sandfly bite (promastigote)	[49,52]
*Trypanosoma cruzi*	Protozoa (Kinetoplastida)	Obligate/ Facultative *	Humans, animals (various cells)	Not applicable in the same way as the definitive host	Triatomine bugs (*Triatoma*, *Rhodnius*, *Panstrongylus*); infection via feces at bite site/mucosa.	[48,51]
*Cryptosporidium parvum*	Protozoa (Apicomplexa)	Obligate	Humans, cattle (sexual stage)	Humans, cattle (asexual stage)	Fecal–oral (water, food, contact); flies may act as mechanical vectors	[45,51]
*Echinococcus multilocularis*	Cestode (tapeworm, helminth)	Obligate	Canids (dogs, foxes)	Rodents, humans (accidental)	Fecal–oral (egg ingestion)	[51,57]

* Although many sources classify Leishmania donovani and Trypanosoma cruzi as obligate intracellular parasites, they are listed here as obligate/facultative because their life cycles also include extracellular stage.

### 2.2. Modes of Transmission in Intracellular Parasitic Infections

Intracellular parasites exhibit a wide range of transmission strategies, shaped by their evolution, host specificity, and ecological niches. Understanding these transmission routes is essential for the control of the disease as well as for effective prevention strategies, especially in resource-limited or endemic regions. The major modes of transmission include vector-borne, fecal–oral, vertical (congenital), and direct contact transmission [58,59].

#### 2.2.1. Vector-Borne Transmission

Recent estimates indicate that there are over 200 million insects for every human on Earth, with around 14,000 species that feed on blood and can spread human pathogens and vector-borne diseases. These diseases cause more than 17% of all infectious diseases worldwide and result in at least 700,000 deaths each year [60,61]. These diseases are called Neglected Tropical Diseases (NTDs) because they hit poor people in subtropical and tropical areas the hardest. Most research has been on diseases spread by mosquitoes, like dengue and malaria. However, other groups of insects, like triatomine bugs, sandflies, and ticks, also have a big effect on the world’s disease burden. More specifically, important vector-borne diseases are:

(i) malaria (*Plasmodium falciparum*, *P. vivax*, *P. ovale*, *P. malariae*, *P. knowlesi*)—spread by *Anopheles* mosquitoes and causing hundreds of thousands of deaths each year.
(ii) Leishmaniasis (*Leishmania* spp.): transmitted by phlebotomine sandflies; it is still one of the most neglected tropical diseases with a high rate of illness and death.
(iii) Chagas disease (*Trypanosoma cruzi*): spread by triatomine bugs; mostly affects Latin America but is being reported more and more in places where it is not common.
(iv) African trypanosomiasis, also known as sleeping sickness (*Trypanosoma brucei gambiense*, *T. b. rhodesiense*): transmitted by tsetse flies (*Glossina* spp.); causes severe neurological disease if untreated.
(v) Babesiosis (*Babesia microti*, *B. divergens*), transmitted by ixodid ticks; significant in humans and animals, particularly in temperate regions.
(vi) Theileriosis (*Theileria parva*, *T. annulata*), transmitted by ticks (*Rhipicephalus*, *Hyalomma*), primarily affects livestock but is significant in veterinary parasitology. Filarial infections, such as lymphatic filariasis caused by *Wuchereria bancrofti*, *Brugia malayi*, and *Onchocerca volvulus*, are transmitted by mosquitoes (*Culex*, *Anopheles*, *Aedes*) or blackflies (*Simulium* spp.) and are significant contributors to chronic morbidity in endemic areas. These vector-borne infections are difficult to control because vectors are ecologically resilient, so we need both medical treatments and integrated vector management strategies [62,63,64,65,66,67,68].

#### 2.2.2. Fecal–Oral Transmission

Protozoan parasites frequently disseminate via the fecal–oral route. It allows infectious stages to move from definitive or intermediate hosts to humans via contaminated food, water, and the environment. This path is important for learning how *Cryptosporidium* spp. and *Toxoplasma gondii* spread. These parasites can cause significant illness in both humans and animals. In *Toxoplasma gondii*, cats and other felids (definitive hosts) eliminate sporulated oocysts through their feces. These oocysts can spread disease in the environment, making it unsafe to eat water, soil, vegetables, and animal feed. People usually get infections by eating sporulated oocysts from fruits and vegetables that have not been washed, drinking dirty water, or eating meat that has not been cooked enough from infected intermediate hosts [69,70,71,72]. *Cryptosporidium* spp. are primarily transmitted via the fecal–oral route, most commonly through the ingestion of water contaminated with oocysts. Waterborne outbreaks are frequent due to the limited efficacy of chlorine-based disinfectants against these oocysts. The parasites produce cysts and oocysts capable of surviving outside the host for extended periods, contributing to their persistence in the environment. This facilitates their transmission via contaminated food, water, and fomites, enabling the parasites to spread across various environments. There have been reports of zoonotic, person-to-person, recreational water, and foodborne transmission pathways, in addition to waterborne transmission. Some researchers have posited that aspiration and hematogenous dissemination may elucidate respiratory cryptosporidiosis in particular instances [73,74,75,76]. *Echinococcus multilocularis* is a cestode parasite that spreads via the fecal–oral route. Dogs, foxes, and other canids serve as hosts, shedding parasite eggs in their feces. These eggs contaminate water sources, vegetation, and animal fur, facilitating environmental transmission. Humans may become accidental intermediate hosts through ingestion of these eggs, often via contact with contaminated food, water, or surfaces. Once ingested, the eggs hatch in the small intestine, initiating the larval stage of the parasite. After that, the oncospheres go through the intestinal wall and into the liver, where they become alveolar hydatid cysts. This indirect fecal–oral transmission is crucial to the epidemiology of alveolar echinococcosis, a severe and often fatal zoonotic disease if untreated [77,78].

#### 2.2.3. Vertical Transmission

Vertical (congenital) transmission is a significant pathway for the spread of different protozoan parasites, involving the passage of infectious stages from an infected mother to her offspring during pregnancy, childbirth, or breastfeeding. This route bypasses environmental stages and enables direct transfer of the pathogen across the placental barrier or during perinatal contact. It plays a major role in the epidemiology of *Trypanosoma cruzi*, *Toxoplasma gondii*, and *Plasmodium falciparum*, all of which can cause severe neonatal and fetal outcomes [79,80,81,82]. In *Toxoplasma gondii*, felids serve as definitive hosts, but vertical transmission occurs when a pregnant woman acquires primary infection during gestation. Tachyzoites disseminate via maternal blood, cross the placenta, and infect the fetus. Clinical outcomes range from subclinical infection to severe congenital toxoplasmosis with chorioretinitis, hydrocephalus, or fetal death. The risk of transmission increases with gestational age, whereas the severity of disease is often greater when infection occurs earlier in pregnancy [83,84,85,86]. Chagas disease (CD), caused by the protozoan parasite *Trypanosoma cruzi*, is recognized by the World Health Organization a neglected tropical disease. Congenital transmission of CD has emerged as an increasingly important public health concern, progressively becoming a predominant route of infection in some regions, both in endemic and non-endemic countries. On the other hand, vertical transmission occurs when bloodstream trypomastigotes cross the placental barrier and invade fetal tissues. Although the majority of congenitally infected newborns are asymptomatic at birth, they have a higher likelihood of prematurity, low birth weight, and reduced Apgar scores compared to uninfected neonates. In more severe cases, clinical manifestations may include hepatosplenomegaly, myocarditis, and even early mortality. The risk and severity of congenital infection are influenced by maternal parasitemia levels, the virulence of the parasite strain, and the immune status of the mother. This highlights the need for systematic maternal screening, early diagnosis in newborns, and timely antiparasitic treatment to prevent or mitigate adverse outcomes [87,88,89,90]. In *Plasmodium falciparum* infection, parasitized erythrocytes adhere to chondroitin sulfate A receptors on the syncytiotrophoblast surface and sequester within the placental intervillous space. This sequestration disrupts placental blood flow and impairs nutrient and gas exchange between the mother and fetus. The resulting condition, known as placental malaria, is associated with intrauterine growth restriction, low birth weight, preterm delivery, and increased perinatal mortality. Vertical transmission of *P. falciparum* occurs more frequently in areas of high malaria endemicity and is often linked to maternal anemia, high-density parasitemia, and inadequate immune protection in primigravidae. Infected newborns may present with congenital malaria, characterized by fever, anemia, and parasitemia in the first days or weeks of life, although some cases remain subclinical at birth [91,92,93]. Neosporosis, a devastating worldwide disease caused primarily by *Neospora caninum* and less commonly by *Neospora hughesi*, is another example of a parasitic infection transmitted via both vertical and horizontal routes. *N. caninum* mainly affects dogs and cattle, occasionally infecting horses, sheep, and deer, while *N. hughesi* is restricted to horses, causing equine protozoal myeloencephalitis. In cattle, neosporosis is frequently associated with congenital transmission, leading to sporadic abortions (~11%). However, abortion rates can rise sharply (30–57%) when infection is acquired during pregnancy. The disease also imposes a substantial economic burden on livestock production worldwide; for example, in New Zealand, *N. caninum*-related reproductive losses represent a major agricultural cost. The ability of *N. caninum* to exploit multiple transmission pathways underscores the epidemiological complexity of certain protozoan infections and the need for integrated control measures [94,95]. Preventive measures focus on routine maternal screening in endemic areas, timely treatment upon detection of infection, and minimizing exposure to infectious sources during pregnancy.

#### 2.2.4. Direct Contact and Sexual Transmission

Although rare among intracellular parasites, certain bacterial species such as *Chlamydia trachomatis*, an obligate intracellular pathogen, are transmitted through direct mucosal contact as well as by sexual activity. *C. trachomatis* causes a range of urogenital and ocular infections and is one of the most common sexually transmitted infections worldwide [96]. Other rickettsial bacteria may also be transmitted through contact with infected tissues or blood.

#### 2.2.5. Environmental and Foodborne Routes

In addition to fecal–oral and vector-borne routes, foodborne transmission plays a significant role in the spread of intracellular parasites. For instance, the consumption of undercooked lamb, pork, or wild game harboring tissue cysts of *T. gondii* is a major infection route in developed countries. Moreover, *Coxiella burnetii*, the agent of Q fever, can be transmitted through inhalation of contaminated aerosols from infected animals or consumption of unpasteurized milk [97,98,99].

## 3. Advances in Diagnostics, Control Strategies, and Vaccine Development for Intracellular Parasites

### 3.1. Advances in Diagnostics

Recent years have seen significant improvements in diagnostic technologies for intracellular protozoan infections, such as *Leishmania* spp., *Toxoplasma gondii*, and *Plasmodium* spp., focusing on enhancing specificity, sensitivity, and accessibility, especially in resource-limited settings. Molecular methods, including loop-mediated isothermal amplification (LAMP), quantitative PCR (qPCR), and multiplex PCR, have revolutionized pathogen detection by enabling quick, highly sensitive identification and simultaneous detection of multiple species. qPCR remains the laboratory-based ‘gold standard’ due to its quantitative accuracy, while LAMP offers a field-deployable alternative that requires minimal equipment. Notably, the LAMP-MS microchip platform, using Chelex-100/boiling extraction, achieved 97.5% sensitivity for the pan-*Plasmodium* target and 100% sensitivity for the *P. falciparum* target; 5% sensitivity for the pan and 94% for the *Pv* target in *P. vivax* samples; and 100% specificity with no false positives in 100 non-infected samples [100,101,102,103]. Serological assays, including ELISA (Enzyme-Linked Immunosorbent Assay) and IFAT (Indirect Fluorescent Antibody Test), remain widely used for surveillance and diagnosis, particularly in chronic infections. They can detect antibodies or antigens but are limited by cross-reactivity and their inability to distinguish past from active infections [104,105]. In malaria diagnostics, rapid diagnostic tests (RDTs) that target antigens such as histidine-rich protein 2 (HRP2) or lactate dehydrogenase (LDH) provide point-of-care solutions. However, their performance varies depending on parasite density and genetic factors. In Cameroon, deletions in pfhrp2 and pfhrp3 genes, especially double deletions (pfhrp2–/pfhrp3–), were responsible for up to about 80% of false-negative RDT results. Conversely, in Timika, Papua (Indonesia), the prevalence of pfhrp2 deletion was low (about 2%, 95% CI 1.10–3.67%), suggesting that HRP2-based RDTs remain largely suitable there [106,107,108]. Toxoplasmosis (caused by the already mentioned *Toxoplasma gondii*) has traditionally been diagnosed using serological assays such as the Dye Test and the Modified Agglutination Test (MAT), which have long served as the ‘gold standard’ for detecting IgM and IgG antibodies. However, serology has well-recognized limitations, particularly reduced sensitivity in the early stages of infection, false-negative results in immunocompromised patients, and diagnostic challenges in newborns due to passive transfer of maternal IgG. These constraints have driven the increasing adoption of molecular diagnostic approaches [109,110]. The most widely applied molecular tools include real-time PCR, nested PCR, semi-nested PCR, and PCR–RFLP, which typically target highly repetitive genomic elements such as the B1 gene (~35 copies) and the 529 bp repetitive element (~250 copies). Semi-nested PCR assays targeting the b1 gene have been shown to detect congenital toxoplasmosis earlier than serology. Nevertheless, studies in newborns have reported notable discrepancies between PCR and serological results, with PCR-positive cases testing seronegative for IgM and IgG. Such findings suggest the possibility of hypersensitivity or false positives, underscoring the need for combined diagnostic approaches integrating molecular assays with serology and clinical evaluation. Real-time PCR technologies, particularly SYBR Green and TaqMan probe assays, provide both quantitative and qualitative detection, enabling parasite burden estimation and treatment monitoring. Evidence indicates that real-time PCR assays targeting the 529 bp (base pair) repetitive element outperform B1-targeted assays in sensitivity, due to the higher copy number of the target sequence [111,112,113,114]. Importantly, leishmaniasis is a neglected tropical disease where traditional parasitological diagnosis (microscopy, culture) remains the reference standard but suffers from low sensitivity, delaying treatment. Molecular methods, particularly PCR-based assays, provide superior sensitivity and specificity, enabling species identification. A novel real-time PCR targeting the conserved Hsp20 gene demonstrated high performance with SYBR Green detection (sensitivity 88%, specificity 100%), surpassing the same assay with TaqMan probes (sensitivity 47%). Isothermal methods, such as loop-mediated isothermal amplification (LAMP) targeting 18S rDNA and kDNA, achieved up to 95% sensitivity for cutaneous and 92% for visceral leishmaniasis, with near-perfect specificity, using minimally invasive samples. These advancements, alongside emerging techniques like recombinase polymerase amplification and portable PCR systems, offer rapid, accurate, and field-adaptable tools, crucial for early case detection and control in endemic areas [115,116,117,118,119]. *Echinococcus multilocularis* is a life-threatening zoonosis where conventional imaging remains the reference standard but often fails to distinguish early lesions from malignancies. Serological tests (ELISA, Western blot using Em2, Em18, EgB antigens) show high sensitivity and specificity (>90–95%) yet cannot reliably differentiate past exposure from active disease and are less effective in immunocompromised patients. Molecular approaches, particularly qPCR targeting multicopy elements, provide superior accuracy and represent the current laboratory ‘gold standard’, while LAMP enables rapid field detection. Real-time PCR on circulating cell-free DNA offers near-perfect specificity (~99%) but moderate sensitivity (~50%), underscoring the need for combined diagnostic strategies. Emerging tools, including proteomic profiling and recombinant antigen–based RDTs, hold promise for point-of-care application and earlier detection of alveolar echinococcosis in clinical and epidemiological settings [120,121,122].

### 3.2. Control Strategies

**Figure 1:**
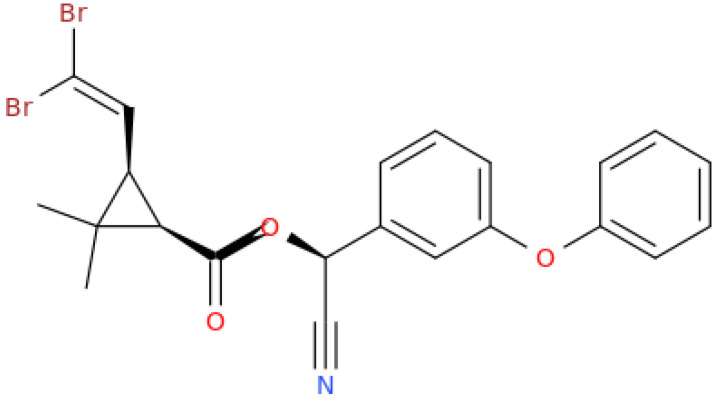
Structural representation of deltamethrin, a pyrethroid insecticide used in IRS programs to reduce sandfly density and cutaneous leishmaniasis incidence.

Effective control of intracellular parasitic diseases relies on an integrated, evidence-based approach that combines environmental modifications, vector management, targeted chemotherapy, and community engagement. The WHO Global Vector Control Response 2017–2030 outlines that coordinated interventions, especially those integrating vector control with surveillance and insecticide resistance management, can substantially reduce disease burden, with global targets of ≥75% reduction in mortality and ≥60% reduction in incidence by 2030 compared with 2016 levels [123,124,125,126]. Integrated Vector Management (IVM) employs measures such as larviciding, long-lasting insecticidal nets (LLINs), insecticide-treated nets (ITNs), indoor residual spraying (IRS), and environmental interventions to reduce vector breeding sites. Cochrane meta-analyses indicate that ITNs lower all-cause child mortality by 17–20% and clinical malaria episodes by ~40–50%, while LLINs also reduce sandfly densities, indirectly protecting against *Leishmania* transmission. IRS with pyrethroids (e.g., deltamethrin, Figure 1) has been shown to decrease indoor sandfly density by 40–80% and cutaneous leishmaniasis incidence by 40–68% [127,128,129].

### 3.3. Vaccine Development

The development of vaccines against intracellular parasites (*Toxoplasma gondii*, *Plasmodium falciparum*, *Leishmania* spp.) remains a major scientific and public health challenge due to their complex life cycles, marked antigenic diversity, and advanced immune evasion strategies, all of which constitute obstacles to the formation of stable, long-lasting immunity. Nevertheless, progress in the past decade has provided hope, with some vaccines reaching late clinical phases and even being introduced in endemic countries [130]. Given the critical importance of vaccination and prevention and mortality reduction, the following section reviews currently available vaccines, ongoing research, and the historical trajectory of their development.

#### 3.3.1. *Plasmodium falciparum* (Malaria)

The first successful malaria vaccine to complete large-scale clinical evaluation was Mosquirix, developed through research initiated in the late 1980s by GlaxoSmithKline (GSK) in partnership with the PATH Malaria Vaccine Initiative, with support from the Bill & Melinda Gates Foundation [131,132,133,134]. The vaccine targets the circumsporozoite protein (CSP) of *P. falciparum*, aiming to block infection at the pre-erythrocytic stage before the parasite enters the bloodstream following a mosquito bite. Early Phase I human trials in the 1990s demonstrated promising results, while large-scale Phase III trials (2009–2014) across seven African countries recorded approximately 55% efficacy against clinical malaria in the first year after the three-dose primary series. However, efficacy waned over time, dropping to ~36% after four years without a booster. The addition of a fourth dose at 20 months significantly improved long-term protection, leading to sustained reductions in severe malaria and all-cause mortality. RTS, S/AS01 was formally recommended by the WHO in October 2021 for use in children under five years of age in moderate- to high-transmission areas [135,136]. Pilot implementation programs in Kenya, Ghana, and Malawi confirmed real-world effectiveness, showing approximately 13% reduction in all-cause mortality and about 31% reduction in severe malaria hospitalizations among vaccinated children. The four-dose schedule (0, 1, 2, and 20 months) has demonstrated a consistent safety profile and sustained protection through the fourth year of life [137,138,139]. A major recent milestone is the development of R21/Matrix-M™, created by the University of Oxford in collaboration with the Serum Institute of India. This vaccine contains a higher density of CSP antigen and employs Novavax’s Matrix-M saponin-based adjuvant to enhance immunogenicity. WHO recommended R21 in October 2023, and it was prequalified in December 2023, enabling global procurement via Gavi and UNICEF. Phase III trials in seasonal transmission settings reported 72–75% efficacy in the first year, with protection maintained above 70% after a booster dose. The affordability of the proposed dose range supports large-scale deployment. By April 2025, over 13 million doses had been delivered, and 19–20 African countries had included the vaccine in their national programs; for example, South Sudan received 645,000 doses in May 2024 for its first rollout [140,141,142]. In addition to these two licensed vaccines, research is progressing on next-generation candidates and monoclonal antibodies that aim to complement pre-erythrocytic vaccines by reducing disease severity and disrupting transmission. Notable examples include RH5.1/Matrix-M, which targets the blood-stage antigen RH5 to prevent erythrocyte invasion and the transmission-blocking monoclonal antibody TB31F, directed against the Pfs48/45 gametocyte antigen [143,144,145].

#### 3.3.2. *Leishmania* spp. (Leishmaniasis)

**Figure 2:**
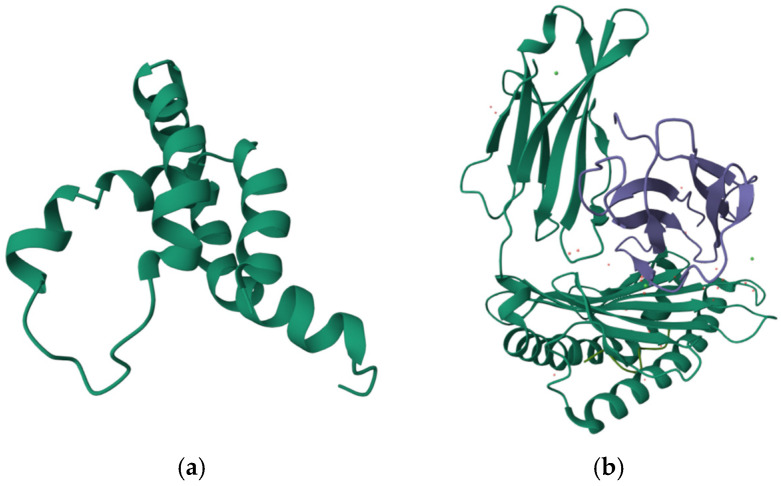
Three-dimensional NMR structure of Kinetoplastid Membrane Protein 11 (KMP11, (**a**)) from *Trypanosoma brucei brucei* (available at the link https://www.rcsb.org/3d-view/5Y70/0, accessed on 2 September 2025) [149] and (**b**) the *Cryptosporidium parvum* gp40/15-derived epitope presented by the murine MHC class I molecule H-2Kb (available at the link https://www.rcsb.org/3d-view/7WCY/1, accessed on 2 September 2025) [150].

The first genetically engineered live-attenuated *Leishmania* vaccine candidates to advance toward clinical development were centrin gene-deleted parasites (LmCen^−^/^−^, LmexCen^−^/^−^), generated using CRISPR/Cas9 technology without antibiotic resistance markers. Research on LmCen^−^/^−^ was initiated in the 2010s by the US Food and Drug Administration (FDA) and academic collaborators, building on decades of studies with attenuated parasites. Preclinical studies in mice and hamsters demonstrated that LmCen^−^/^−^ immunization is safe, does not provoke lesions, and ensures long-term cross-protection against both cutaneous (*L. Mexicana*, *L. major*) and visceral (*L. donovani*) challenge, including sand fly-mediated transmission. Protective efficacy is associated with increased IFN-γ/IL-10 and IFN-γ/IL-4 ratios, indicating a durable Th1 immune response. In parallel, the LmexCen^−^/^−^ strain, developed for New World cutaneous leishmaniasis, has shown cross-protection against visceral leishmaniasis in hamsters, significantly reducing spleen and liver parasite burdens and improving survival after *L. donovani* challenge [146,147,148]. Other advanced candidates include the ChAd63-KH viral-vectored vaccine, encoding kinetoplastid membrane protein 11 (KMP-11, Figure 2) and hydrophilic acylated surface protein B (HASPB), which has completed Phase I/II trials in the UK and Sudan.

These studies reported strong immunogenicity, safety, and CD8^+^ T-cell induction, with Phase 2 trials for visceral leishmaniasis ongoing in Sudan [151,152,153]. Veterinary vaccine development against canine visceral leishmaniasis (CVL) has resulted in several licensed products: CaniLeish^®^ (purified LiESP/QA-21 adjuvant, EU), Leish-Tec^®^ (recombinant A2 antigen + saponin, Brazil), and LetiFend^®^ (chimeric QLet1 protein, EU), each targeting distinct recombinant or purified antigens. While these vaccines reduce canine disease burden, publicly available data and regulatory summaries indicate that their effect on *Leishmania* transmission to humans remains unconfirmed. Notably, Leish-Tec^®^ production and distribution were suspended in 2023 following non-compliance with licensed A2 antigen content [154,155]. Dog is an appropriate model to objectively evaluate the effectiveness of a vaccine targeted for ZVL. A small number of vaccine candidates have been tested in dogs, and four vaccines have obtained a commercial license against CVL: (i) Leishmune^®^ in Brazil, a semi-purified fucose–mannose ligand antigen (FML) adjuvanted with Quil-A^®^. However, the Leishmune^®^ license has been suspended since 2014 as the vaccine did not fulfil the phase III requirements in terms of vaccine efficacy; (ii) Leish-Tec^®^, the only vaccine currently sold in Brazil, which contains a recombinant protein A2 adjuvanted with saponin; (iii) CaniLeish^®^ in Europe, composed of *L. infantum* excreted/secreted products (LiESAp) and adjuvanted with QA-21; and finally, (iv) LetiFend^®^, which contains Protein Q as active ingredient, has received marketing authorization by the European Commission. LetiFend^®^ is a recombinant chimeric protein vaccine composed of immunodominant peptide sequences (QLet1) from *L. infantum* antigens, making it a peptide-based formulation. This differentiates it from live-attenuated or viral-vectored vaccines by presenting only selected peptide epitopes to elicit protective immunity [156,157,158,159,160]. Recent CVL vaccine research has increasingly prioritized peptide and other subunit strategies, especially chimeric and multi-epitope constructs, because they enable safer profiles than live organisms, rational epitope selection for strong Th1-skewed T-cell responses, and scalable, quality-controlled manufacturing. By the way, to date, he only commercial chimeric protein vaccine remains LetiFend^®^ (QLet1), authorized across the EU for reducing the risk of *L. infantum* disease in dogs [161,162,163]. Experimental chimeric candidates continue to show promise: for example, ChimT formulations have significantly reduced parasite burdens and induced protective Th1 immunity in murine models, and systematic reviews report robust parasite-load decreases across chimeric/multi-epitope subunit platforms [164,165]. Complementary approaches also advance the field: a centrin-deleted live-attenuated LmCen^−^/^−^ vaccine reached ~82% efficacy in naturally exposed dogs in Tunisia, highlighting zoonotic control potential, while vector-saliva–based vaccines such as PdSP15 have conferred protection in non-human primates under sand-fly–transmitted challenge [166,167]. Despite these advances, no licensed human vaccine exists for any form of leishmaniasis. Key challenges include eliciting durable, cross-species immunity, standardizing correlates of protection, and conducting large-scale, multicountry field trials in diverse endemic settings. Promising human vaccine platforms currently in development integrate live-attenuated strains, viral vectors (e.g., the already cited ChAd63-KH), and multi-antigen protein formulations with modern adjuvants, as well as mRNA vaccine candidates entering preclinical evaluation. Furthermore, the establishment of a Controlled Human Infection Model (CHIM) for cutaneous leishmaniasis in 2024 offers a powerful tool for accelerating early-phase human vaccine assessment [168,169,170].

#### 3.3.3. *Toxoplasma gondii*

The development of an effective vaccine against *Toxoplasma gondii* remains one of the central challenges in modern parasitology. To date, no licensed human vaccine exists, while in veterinary medicine, only Toxovax^®^, based on the attenuated S48 strain, is available and used exclusively for the prevention of congenital toxoplasmosis in sheep. In recent years, considerable efforts have been directed toward novel experimental platforms. Among the most promising are genetically attenuated live vaccines, such as WH3 Δrop18, which in murine models have induced strong cell-mediated and humoral immune responses, including elevated IFN-γ, IL-12, TNF-α, activation of CD4^+^ and CD8^+^ T lymphocytes, and production of specific IgG antibodies. Vaccinated mice displayed high survival rates upon challenge with multiple *T. gondii* strains (RH, ME49, WH3, WH6), while brain cyst burden was markedly reduced [171]. Another promising direction involves multi-epitope peptide vaccines, designed through in silico prediction of immunogenic epitopes from antigens such as GRA5, GRA6, SAG1, and ROP18. In HLA-A*11:01 transgenic mice, a polypeptide construct combining several CD8^+^ epitopes and the universal CD4^+^ epitope PADRE with the adjuvant GLA-SE elicited expansion of CD8^+^ memory pools, robust T-cell responses, and a significant reduction in brain cysts after challenge (up to 80% survival) [172]. mRNA vaccines have also emerged as an innovative strategy, with the TG290 mRNA-LNP formulation inducing both humoral (IgG1/IgG2a) and cellular responses, by activating helper and cytotoxic T lymphocytes and extending survival in infected animal models [173]. In parallel, nanoparticle-based delivery systems (chitosan, PLGA, lipid nanoparticles) have demonstrated the ability to stimulate dendritic cell activation, enhance antigen presentation, and promote Th1/Th17-type immune responses, offering a safe and versatile platform for vaccine design [174]. Additional approaches include subunit and DNA vaccines, which utilize immunodominant proteins from the GRA, SAG, and MIC families. These platforms provide a high safety profile, but their protective efficacy in animal models remains partial and requires optimization of antigen combinations and adjuvant systems [175]. Innovative alternatives such as gamma-irradiated tachyzoites and exosome-based vaccines have also been explored, aiming to combine humoral and cellular immune mechanisms, although they are still at an early stage of development. Overall, progress in vaccine research underscores that the major challenges remain the achievement of broad cross-strain protection, the induction of durable cell-mediated immunity, and the establishment of scalable production strategies. Insights gained from peptide-based, live-attenuated, and mRNA platforms indicate that innovative delivery systems and multi-antigen constructs will be central to the design of the next generation of effective and safe vaccines against *Toxoplasma gondii*. In this context, the most promising scenario for a clinically viable human vaccine is likely to involve hybrid approaches that integrate epitope-based peptides with advanced delivery technologies and potent adjuvants, providing both safety and long-lasting cross-protective immunity [168,176,177].

#### 3.3.4. *Echinococcus multilocularis*

In recent years, vaccination strategies against *Echinococcus multilocularis* have primarily focused not on humans but on intermediate hosts such as sheep, rodents, and dogs. Targeting these hosts can effectively disrupt the parasite’s transmission cycle and thereby reduce the risk of human infection. A number of recombinant antigens, including EmY162, Em95, and Eg95, have been tested in animal models, demonstrating high levels of protective immunity and significantly limiting parasite development. These findings indicate that recombinant antigen-based vaccines represent a promising approach for immunoprophylaxis in intermediate hosts. In parallel, research has also explored the potential of DNA vaccines, particularly plasmid constructs encoding emY162 and related genes. Experimental studies in rodents have shown that these vaccines elicit a Th1-oriented protective immune response, characterized by cytokine-driven activation that prevents parasite proliferation [178,179].

#### 3.3.5. *Cryptosporidium* spp.

Vaccination strategies against *Cryptosporidium* spp. have primarily focused not on humans but on livestock hosts, particularly neonatal calves. Targeting these hosts can effectively disrupt the parasite’s transmission cycle and reduce zoonotic risk. Several recombinant antigens and monoclonal antibody–based platforms, such as Cp23 and Gp40, have been tested in animal models, showing high levels of protective immunity and significant reductions in oocyst shedding. These findings indicate that antigen-based vaccines represent a promising approach for immunoprophylaxis in veterinary medicine [180,181]. In parallel, research has also explored the potential of DNA and mRNA vaccines, particularly plasmid constructs encoding immunodominant *Cryptosporidium* antigens. Experimental studies in murine and bovine models have demonstrated that these vaccines elicit a Th1-oriented immune response, characterized by cytokine-driven activation that limits parasite proliferation. Recent veterinary approvals of BOVILIS CRYPTIUM (tradename for a vaccine based on *Cryptosporidium parvum* glycoprotein gp 40, Figure 1) in the EU (2023) and UK (2024), and its clearance in the US (2025), underscore the translational value of such approaches in calves [182,183]. Despite encouraging progress in animal models, several challenges remain. No licensed vaccine currently exists for human use, and the development of such a vaccine is hampered by the parasite’s complex life cycle and the tumor-like growth of alveolar cysts. These biological features complicate both the efficacy and safety of vaccine candidates, underscoring the need for integrated, multi-target strategies that combine immunological, molecular, and ecological interventions [184,185,186].

In this context, Table 2 summarizes currently available and experimental vaccines or vaccine candidates against key intracellular parasites (*Plasmodium falciparum*, *Leishmania* spp., *Toxoplasma gondii*, *Trypanosoma cruzi*, *Cryptosporidium parvum*, and *Echinococcus multilocularis*). In particular, the same table highlights their platforms, antigenic targets, stage of development, geographic scope, and efficacy, providing a comparative overview of advances in parasitic vaccine research.

## 4. Intracellular Parasitic Infections in Armenia: Epidemiological Trends, Diagnostic Gaps, and Future Directions Within a ‘One Health’ Framework

**Figure 3:**
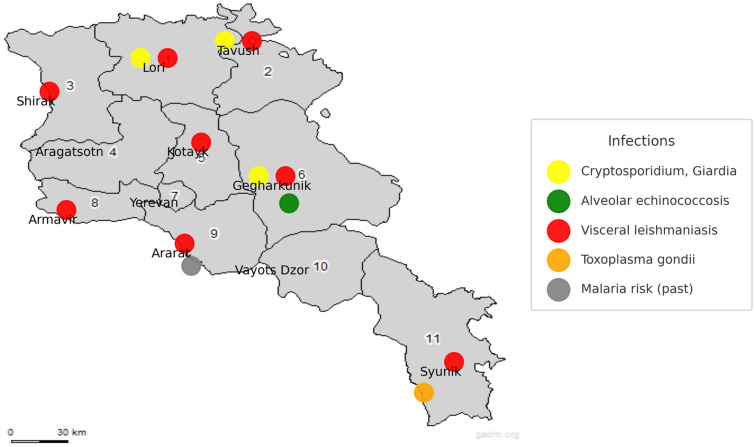
Provincial distribution of major parasitic infections in Armenia (*Cryptosporidium*, *Giardia*, *Echinococcus multilocularis*, *Leishmania infantum*, *Toxoplasma gondii*, and historical malaria risk). The map was generated by the use of shapefiles available from https://gadm.org/download_country_v3.html (accessed on 13 October 2025).

Recent estimates suggest that more than 45% of infectious disease cases reported in Armenia are attributable to parasitic pathogens; however, this figure is likely underestimated due to the absence of systematic surveillance and limited diagnostic capacity. Armenia, an ecologically diverse and socio-politically evolving country in the South Caucasus, presents a unique setting for studying intracellular parasitic infections. Systematic research on infections caused by *Leishmania* spp., *Toxoplasma gondii*, and *Plasmodium* spp. remains limited in the region. The spatial distribution of key parasitic infections and risk areas in Armenia is shown in Figure 3.

Considering the mountainous terrain, semi-closed rural communities, lack of effective control mechanisms, and potential vector expansion amid climate change, Armenia is a logical and necessary case study for the epidemiological assessment of these infections. Considering the public health importance of parasitic infections and the extensive research conducted worldwide, increasing attention has also been paid in recent years to studies carried out in Armenia [210,211]. This is particularly relevant given the scarcity of local data, and such findings enable valuable comparisons with international research outcomes. Data on waterborne protozoa in Armenia remain very limited compared to global investigations. The few available studies, however, suggest that *Cryptosporidium* and *Giardia* are present in both human populations and environmental reservoirs. The last reported investigation of human cryptosporidiosis was conducted in 2011 in Vanadzor, Armenia’s third largest city. Using modified Ziehl–Neelsen (mZN) staining and ELISA, prevalence was estimated at 7% (mZN) and 17% (ELISA) among patients with gastrointestinal complaints, with infections peaking in September. Comparable data on human giardiasis is lacking [211]. Animal surveys provide further insight, with the first livestock survey (2006) reporting *Cryptosporidium* in 23% of sheep, 31% of cattle, and 50% of pigs, particularly in younger animals. A second survey (2012) confirmed high occurrence in four regions and Yerevan suburbs: 22% of lambs, 25% of calves, and 20% of piglets, with no detection in rabbits or chickens; prevalence was highest in autumn [212]. No data on *Giardia* infection in animals is currently available. More recent environmental monitoring (2019–2024) revealed that both *Cryptosporidium* oocysts and *Giardia* cysts occur widely in Armenian water bodies. In fact, of five samples tested specifically for *Giardia* (three water, two sediments), four were positive. Particularly high parasite loads were detected in sediment samples, implying continuous contamination and accumulation in riverbeds. For example, in the area near the river Aghstev, located near Ijevan (~18,000 inhabitants) and downstream of Dilijan (~30,000 inhabitants), untreated sewage discharge correlated with high contamination levels. One water sample from Lake Sevan, collected in a recreational zone with more than 100 daily summer visitors, tested positive for *Cryptosporidium* by both mZN and qPCR despite relatively low oocyst counts [212]. These findings underscore significant data gaps and methodological challenges. Limitations include small sample sizes, heterogeneous diagnostic methods (IFAT, qPCR, mZN, ELISA), lack of PCR internal controls, and no recovery efficiency assessments. Nevertheless, preliminary results confirm that waterborne protozoan parasites are present and likely pose a risk to public health in Armenia, emphasizing the need for systematic monitoring of sediments, water, humans, and animals. Other parasitic diseases of zoonotic origin, particularly helminthic infections, have also been documented in Armenia in recent decades. Alveolar echinococcosis (AE), caused by *Echinococcus multilocularis*, was traditionally considered non-endemic; however, recent investigations have identified human AE cases over the past decade. Most were detected among patients undergoing liver surgery and originated largely from rural areas, especially the Gegharkunik region. The average annual incidence was approximately 0.033 per 100,000 population, peaking at 0.10 in 2017 [37]. Interestingly, a retrospective case-finding investigation conducted between 2008 and 2020 identified 11 confirmed human AE cases, corresponding to an estimated annual incidence of 0.033 per 100,000 inhabitants. Case confirmation relied on tissue biopsy and advanced medical imaging. The affected individuals were aged 12–58 years (median 33 years), notably younger than typical AE cohorts in Western Europe. More than half of the cases (6/11; 55%) originated from rural communities in Gegharkunik Province, an area characterized by close human–animal contact and environmental exposure conducive to parasite transmission. The estimated annual incidence fluctuated between 0.032 per 100,000 in 2008 and a peak of 0.1 per 100,000 in 2017. These values are comparable to those reported from well-documented endemic European countries, including Switzerland and Germany. Most Armenian patients were diagnosed at advanced hepatic stages, underscoring substantial delays in case recognition and the absence of systematic surveillance. These findings not only highlight the underestimated burden of AE in Armenia but also suggest the need for enhanced national monitoring programs, standardized diagnostic guidelines, and awareness campaigns, particularly in rural high-risk regions [213,214]. In addition to waterborne protozoa (*Cryptosporidium*, *Giardia*) and zoonotic helminths such as *Echinococcus multilocularis*, Armenia is also at risk from vector-borne protozoan parasites. Among these, visceral leishmaniasis (VL) represents one of the most significant threats, given its history of disappearance and subsequent re-emergence in the country. VL was first described in Armenia in 1913. During the following decades, the number of cases steadily increased, and between 1935 and 1969, a total of 821 infections were officially reported, mainly in children. After 1969, no new cases were registered, suggesting that the disease had been eliminated. However, this success proved temporary as, following a 30-year hiatus, VL resurfaced in 1999 with a new autochthonous case that was detected in a four-year-old child [215]. Since its reappearance, VL has once again established a foothold in Armenia. Between 1999 and 2016, surveillance documented 116 locally acquired cases, distributed across 8 of the country’s 11 administrative districts. By 2019, the cumulative number had risen to 167 confirmed cases. Despite these records, the actual burden is likely underestimated due to limited laboratory capacity, diagnostic challenges, and insufficient medical awareness [39,201]. The molecular era of VL diagnosis in Armenia began in 2012–2016, when PCR and sequencing confirmed *Leishmania infantum* as the causative agent. In one study, 22 of 25 suspected patients were confirmed positive by microscopy and PCR, with ITS1-PCR-RFLP genotyping providing the first definitive molecular identification of the parasite in the country.

Recent genetic studies have confirmed the circulation of the *L. infantum* MON-1 genotype in Armenia, one of the globally predominant strains. The analyzed strains were compared with regional isolates using PCR-RFLP and microsatellite markers, enabling differentiation between imported and endemic cases [216]. The main vector is the *Phlebotomus* sand fly, widely distributed in Armenia’s forested and mountainous areas. The likely transmission cycle includes domestic dogs as well as wild mammals. Owing to human encroachment into new areas and expanding urbanization, the risk of infection is increasing. Currently, no licensed vaccine exists for humans; however, second-generation DNA vaccines are under active development. Their implementation in Armenia would require localized clinical trials to assess safety and efficacy [217]. This advance marked an important step toward integrating modern diagnostic techniques into national health infrastructure. Taken together, these findings illustrate that VL in Armenia is a re-emerging zoonotic disease, sustained by local sandfly vectors and ecological conditions favorable for transmission. Ongoing surveillance, molecular confirmation, and capacity-building in healthcare settings remain essential to ensure early detection and effective control [216,217,218]. Research on *Toxoplasma gondii* in Armenia has historically been limited and often fragmented, yet recent studies have begun to provide molecular evidence of its circulation in both wild and synanthropic hosts. Early investigations, mostly in Russian- or Armenian-language sources, were restricted to serological surveys in women of reproductive age and livestock, indicating the presence of toxoplasmosis but lacking systematic nationwide coverage [219,220,221]. In Armenia, the seroprevalence of *Toxoplasma gondii* appears relatively high, linked to dietary habits (insufficient thermal processing of meat) and the large population of domestic cats. Primary transmission routes are oral, via oocyst-contaminated food and water or tissue cysts in undercooked meat, and congenitally from mother to fetus [219]. Famously, pregnant women represent a high-risk group. Although no human vaccine is available, veterinary vaccines (e.g., for sheep) exist and may help reduce infection in agricultural settings. Prevention relies on food-safety measures (proper cooking of meat, safe water), public awareness, and hygienic practices (handwashing after gardening/soil contact, safe handling of cat litter, avoiding feeding cats raw meat). The first molecular detection of *T. gondii* infection in wild birds was conducted in the Meghri region of Syunik Province during the breeding seasons of 2013, 2014, and 2018. Blood samples of 116 passerines were tested using PCR with primers derived from the RE gene, revealing a mean prevalence of 12%. The highest infection rates were reported in Upcher’s warbler (*Hippolais languida*) at 36%, eastern black-eared wheatear (*Oenanthe melanoleuca*) at 33%, and eastern Orphean warbler (*Curruca crassirostris*) at 19%. Long-distance migrants had significantly higher infection rates than resident birds (χ^2^ = 7.11, DF = 2, *p* = 0.029), while sex and age were not associated with infection. These findings emphasize the epidemiological role of migratory birds in parasite dissemination across regions intersected by major flyways [210]. Complementary data emerged from a 2024 molecular study on small mammals, which serve as critical reservoirs in the life cycle of *T. gondii*. PCR targeting a 529 bp non-coding fragment of the parasite genome revealed *T. gondii* DNA in 15 out of 137 (10.9%) rodents and shrews captured across six Armenian localities. This evidence confirmed that natural reservoirs actively maintain the parasite and contribute to environmental contamination through the definitive hosts, domestic and wild felids [222]. These findings suggest that human exposure is likely significant, especially considering the close contact between cats and humans in rural households. However, no systematic nationwide sero-epidemiological surveys have been performed among humans. The absence of such data represents a major gap, particularly for pregnant women, as congenital toxoplasmosis remains a critical but underrecognized public health threat. Establishing surveillance in risk groups and integrating toxoplasmosis screening into maternal health programs is therefore strongly recommended. Malaria, despite its official elimination in Armenia in 1963, still retains epidemiological relevance. The country experienced a severe epidemic in the 1920s–1930s, followed by rapid improvements in the 1940s–1950s with the establishment of a malaria control department and health services; a campaign that included indoor residual spraying with insecticides culminated in elimination by 1963 [218]. After roughly three decades without transmission, local cases resurfaced in 1994 amid environmental and socio-political upheavals, and imported infections rose markedly in the mid-1990s (195 cases in 1994; 502 in 1995) [223]. Although sustained local transmission is currently absent, competent *Anopheles* vectors are present in some areas, maintaining a theoretical risk of reintroduction. Effective control, therefore, requires active surveillance and targeted risk assessment among migrants and border communities. The RTS, S/AS01 vaccine is deployed in parts of Africa but has no practical application in Armenia, given the lack of endemic malaria foci. By 1998, a total of 1,156 malaria cases had been reported, of which 542 (approximately 47%) originated within the country [223]. Notably, nearly nine out of ten cases that year were concentrated in the Masis district of the Ararat Valley [224]. Thanks to large-scale control efforts coordinated across sectors, the last three cases of local transmission were reported in 2005 [223,224], and in 2011, Armenia was officially certified by WHO as a malaria-free country [223,224,225]. Despite this achievement, vulnerability remains. Favorable climatic conditions at altitudes up to 1200 m, the constant presence of competent *Anopheles* vectors, and cross-border population mobility support the possibility of reintroduction [226]. Armenia’s experience shows that even after a long hiatus, malaria can re-emerge when environmental and social factors coincide. Therefore, continuous vector surveillance, robust diagnostic infrastructure, and rapid response capabilities are essential to prevent future epidemics. The available evidence demonstrates that Armenia hosts a diverse spectrum of intracellular parasitic infections, including waterborne protozoa (*Cryptosporidium*, *Giardia*), zoonotic helminths (*Echinococcus multilocularis*), and vector-borne protozoa (*Leishmania infantum*). Historical experience with malaria further highlights the country’s vulnerability to re-emerging parasitic threats under favorable ecological and social conditions. At the same time, significant diagnostic gaps and the absence of systematic nationwide surveillance continue to hinder accurate burden estimates for these infections. About pathogens not yet present in Armenia, Chagas disease (*Trypanosoma cruzi*) has never been reported, and there are no known endemic triatomine vectors capable of sustaining transmission. For now, it does not represent a public health concern in the country. Nevertheless, globalization and increased human migration demand vigilance to ensure preparedness for potential imported cases. The main points discussed in this section on key parasitic infections in Armenia, including pathogen types, affected hosts, prevalence, diagnostic methods, and study timelines, are summarised in the following table (Table 3).

Diagnostic capacity in Armenia has improved in recent years with the introduction of molecular methods, including PCR confirmation of *Leishmania infantum* and *Toxoplasma gondii* in animal and environmental reservoirs. However, the diagnostic landscape remains fragmented, relying on heterogeneous methodologies and lacking standardized protocols. Similarly, no vaccines are currently available or under trial in Armenia for parasitic infections. Globally, vaccine development against *Leishmania*, *Toxoplasma*, *Cryptosporidium*, and *Trypanosoma cruzi* remains experimental, with only limited veterinary applications.

Looking ahead, several priorities emerge for Armenia:

(i) establishment of systematic surveillance programs for waterborne, vector-borne, and zoonotic parasites.
(ii) expansion of molecular diagnostic infrastructure to improve early detection and case confirmation.
(iii) awareness and training among healthcare professionals to reduce underreporting and misdiagnosis.
(iv) participation in international research collaborations on parasite epidemiology, molecular typing, and vaccine development.

By adopting an integrated ‘One Health’ approach, Armenia can strengthen its resilience against both endemic and potentially imported intracellular parasitic infections. This strategy will ensure that the country not only addresses present diagnostic and surveillance gaps but also proactively prepares for future parasitological challenges.

## 5. Conclusions

Considering the global importance of parasitic diseases, the development of effective vaccines remains a critical but largely unmet challenge. While malaria has seen notable advances with the introduction of RTS, S/AS01 and R21/Matrix-M, vaccines against other intracellular parasites often remain at preclinical or veterinary stages, reflecting both biological complexity and the need for integrated control strategies. In Armenia, the historical elimination of malaria and subsequent WHO certification as “malaria-free” demonstrate the effectiveness of coordinated action, yet the risk of reintroduction persists alongside surveillance gaps. Recent studies confirm the circulation of waterborne protozoa (*Cryptosporidium*, *Giardia*), the re-emergence of leishmaniasis, and documented echinococcosis cases, emphasizing the need for national monitoring, strengthened molecular diagnostics, and intersectoral collaboration. Adapting international experience to Armenia’s specific epidemiological context is therefore essential, not only to ensure sustainable preparedness against re-emerging and neglected parasitic infections, but also to guide future vaccine development and deployment strategies tailored to regional priorities.

## Figures and Tables

**Table 2:** Current and experimental vaccines or vaccine candidates against selected protozoan and helminth parasites: platforms, targets, clinical status, and efficacy.

Parasite	Vaccine/Candidate	Platform Adjuvant	Antigen Target	Stage Status	Geography	Efficacy	Ref.
*Plasmodium falciparum*	RTS,S/AS01 (Mosquirix)	Protein subunit + AS01	CSP	Phase IV licensed	Sub-Saharan Africa	~30–50%	[187]
*Plasmodium falciparum*	R21/Matrix-M	Protein subunit + Matrix-M	CSP	Phase III Advanced trial	West Africa	>75%	[188]
*Plasmodium falciparum*	PfSPZ Vaccine (Sanaria)	Live attenuated sporozoites	Whole sporozoites	Phase II/III Experimental	Africa, USA	High (under study)	[189]
*Leishmania* spp.	Letifend^®^ (dogs)	Recombinant protein	Q proteins (*L. infantum*)	Licensed (vet) Veterinary	Europe	Protective (dogs)	[190]
*Leishmania* spp.	CaniLeish^®^ (dogs)	Subunit protein vaccine	Excreted secreted protein from *L. infantum*	Licensed veterinary vaccine	Europe	68.4%. ~3.6-fold reduced risk of disease	[191]
*Leishmania* spp.	LeishTec^®^	Recombinant protein vaccine (Quil A/saponin)	rA2 (amastigote antigen A2)	Veterinary licensed	Brazil (endemic zones	80.8% (seroconverted dogs)	[192,193]
*Leishmania* spp.	LEISH-F3 + GLA-SE	Recombinant fusion protein GLA-SE (TLR4 agonist)	NH + SMT fusion	Phase I/II clinical trials		Safe, immunogenic; induced Th1 cytokines; efficacy	[194,195]
*Leishmania* spp.	ChAd63 KH	Viral vector vaccine (chimpanzee adenovirus)	KMP-11 + HASPB (KH antigen)	Phase I clinical trial	UK, Sudan	Safe, strong CD8^+^ responses; efficacy under evaluation	[196]
*Leishmania* spp.	DNA vaccine (gp63 + Hsp70)	DNA plasmid	gp63 + Hsp70	Pre-clinical/experimental	Mouse models	Protective immunity, reduced parasite loads	[197]
*Leishmania* spp.	Multi-epitope peptide vaccine (LACK, LeIF, GP63, SMT)	Synthetic peptides in various formulations	LACK, LeIF, GP63, SMT	Pre-clinical/experimental	Mouse models (India, Iran)	Th1-biased immunity; protection in mice	[198]
*Toxoplasma gondii*	rSAG1	Recombinant protein	SAG1	Preclinical Experimental	Mouse	increased survival	[199]
*T. gondii*	MIC8 + AMA1 heterologous prime/boost	Prime: recombinant vaccinia virus (rVV), Boost: virus-like particles (VLPs)	MIC8 + AMA1 proteins	Preclinical (mouse, BALB/c)—heterologous (rVV + VLP) regimen yields better reduction in brain cysts than VLP + VLP	Preclinical (mouse, BALB/c)	Significant reduction in brain cyst burden compared to homologous VLP + VLP	[200]
*T. gondii*	MIC8 + AMA1 heterologous prime/boost	DNA/protein prime boost varies	MIC8, AMA1	Preclinical (mice)	Lab studies	Protective, experimental	[201]
*T. gondii*	GP–AS04–TE	Glucan particle delivery AS04 (MPL + alum)	*T. gondii* lysate	Preclinical	Lab studies	Improved survival	[202]
*T. gondii*	pVAX1-TgIMC1 nanosphere DNA vaccine	DNA nanosphere Encapsulated delivery	IMC1 protein	Preclinical	Lab studies	Partial protection	[203]
*T. gondii*	SAPN with multi-epitope peptides	Self-assembling protein nanoparticle None/experimental	GRA7 + others	Preclinical	Lab studies	Protective immune response	[204]
*T. gondii*	Chimeric multi-epitope vaccine	DNA/protein varies	SAG1 + apicoplast proteins	Preclinical	Lab studies	Reduced parasite load	[205]
*T. gondii*	Multi-epitope membrane protein vaccine	DNA/protein varies	Predicted membrane epitopes	Preclinical	Lab studies	In silico + mouse testing	[206]
*Trypanosoma cruzi*	multi-epitope recombinant protein	Recombinant chimeric protein (with adjuvant, unspecified)	Trivalent antigen (multi-epitope)	Preclinical Experimental	Animals (mice, others)	Reduced parasite load	[207]
*Cryptosporidium parvum*	Oocyst antigen	Recombinant/purified proteins	Oocyst antigens	Preclinical Experimental	Mice	Reduced shedding	[208]
*Echinococcus multilocularis*	GILE vaccine	Multi-epitope recombinant	EMY162, LAP, GLUT1	Preclinical Experimental	Mice	Reduced cysts	[209]

**Table 3:** Summary of key parasitic infections in Armenia, including pathogen types, affected hosts, prevalence, diagnostic methods, and study timelines.

Pathogen/Disease	Type	Affected Populations/Reservoirs	Prevalence/Incidence	Diagnostic Methods	Timeline	Key Insights/Notes	Ref.
*Cryptosporidium* spp.	Waterborne protozoa	Humans, livestock (sheep, cattle, pigs), environment	Humans: 7% (mZN), 17% (ELISA) in 2011 Animals: Up to 50% in pigs Environment: Widespread (2019–2024)	mZN staining, ELISA (RIDASCREEN^®^), qPCR	2006–2024	Environmental contamination peaks in autumn; high parasite loads in river sediment; linked to untreated sewage (e.g., Aghstev River).	[212]
*Giardia* spp.	Waterborne protozoa	Humans (limited data), environmental samples	4/5 samples positive (2019–2024)	qPCR (environment), no recent human/animal data	2019–2024	High levels in sediment imply long-term contamination; no animal infection data available; human prevalence remains unknown.	[212]
*Echinococcus multilocularis* (AE)	Zoonotic helminth	Humans (esp. rural areas), likely wild canids	11 confirmed cases (2008–2020); peak incidence: 0.1/100,000 in 2017	Tissue biopsy, medical imaging	2008–2020	Previously thought non-endemic; mainly affects younger adults; comparable to endemic European countries; poor early diagnosis.	[37,214]
*Leishmania infantum* (VL)	Vector-borne protozoa	Humans, sandfly vector	167 cases by 2019 (since 1999)	Microscopy, PCR, sequencing (ITS1-PCR-RFLP)	1999–2019+	Re-emerged in 1999 after 30 years; 8/11 regions affected; likely underreported due to diagnostic and awareness gaps.	[29,30]
*Toxoplasma gondii*	Food-/zoonotic protozoa	Birds (migratory), small mammals (rodents, shrews), possibly humans	Birds: up to 36% (PCR); Mammals: 10.9% positive	PCR (RE gene, 529 bp fragment)	2013–2024	Migratory birds aid spread; infection in mammals confirms environmental maintenance; human exposure likely but no national survey exists.	[96,97,98,99]
*Plasmodium* spp. (malaria)	Vector-borne protozoa	Humans (previously), *Anopheles* mosquitoes	1156 cases by 1998; last local case: 2005	Microscopy, epidemiological surveillance	1920s–2011	Eradicated by 1963; resurgence in 1990s due to instability; certified malaria-free in 2011 but remains at risk due to ecological and vector presence.	[223,226]

## Data Availability

Not applicable.

## Abbreviations

The following abbreviations are used in this manuscript:

AE	Alveolar Echinococcosis
bp	Base Pair (unit of DNA length)
CD	Chagas Disease (context-specific abbreviation)
DALY	Disability-Adjusted Life Year
DF	Degrees of Freedom (in statistical tests)
DNA	Deoxyribonucleic Acid
ELISA	Enzyme-Linked Immunosorbent Assay
IFAT	Immunofluorescent Antibody Test
IFN-γ	Interferon-gamma
IgG	Immunoglobulin G
IgM	Immunoglobulin M
IL-10	Interleukin-10
IL-12	Interleukin-12
IRS	Indoor Residual Spraying
IVM	Integrated Vector Management
ITS1-PCR-RFLP	Internal Transcribed Spacer 1-PCR-Restriction Fragment Length Polymorphism
ITNs	Insecticide-Treated Nets
kDNA	Kinetoplast DNA
LLINs	Long-Lasting Insecticidal Nets
mRNA	Messenger Ribonucleic Acid
mZN	Modified Ziehl–Neelsen (staining)
NTDs	Neglected Tropical Diseases
PCR	Polymerase Chain Reaction
qPCR	Quantitative Polymerase Chain Reaction
rDNA	Ribosomal DNA
RE gene	Repetitive Element gene (used in *T. gondii* PCR assays)
RIDASCREEN^®^	Brand name for a commercial ELISA kit
RNA	Ribonucleic Acid
TNF-α	Tumor Necrosis Factor-alpha
VL	Visceral Leishmaniasis
WHO	World Health Organization
χ^2^	Chi-square (statistical test symbol)

## References

[1] Osier F., Uzonna J.E. (2020). Editorial: Regulation of Immunity to Parasitic Infections Endemic to Africa. Front. Immunol..

[2] Ghosh S., Jiang N., Farr L., Ngobeni R., Moonah S. (2019). Parasite-Produced MIF Cytokine: Role in Immune Evasion, Invasion, and Pathogenesis. Front. Immunol..

[3] Mpaka-Mbatha M.N., Naidoo P., Islam M.M., Singh R., Mkhize-Kwitshana Z.L. (2023). Demographic Profile of HIV and Helminth-Coinfected Adults in KwaZulu-Natal, South Africa. S. Afr. J. Infect. Dis..

[4] Lukeš J., Mauricio I.L., Schönian G., Dujardin J., Soteriadou K., Dedet J., Kuhls K., Tintaya K.W.Q., Jirků M., Chocholová E. (2007). Evolutionary and Geographical History of the *Leishmania donovani* Complex with a Revision of Current Taxonomy. Proc. Natl. Acad. Sci. USA.

[5] Maurice A.P., Jenkin A., Norton R.E., Hamilton A., Ho Y. (2020). Epidemiology of Parasitic Diseases.

[6] Torgerson P.R., de Silva N.R., Fèvre E.M., Kasuga F., Rokni M.B., Zhou X.N., Sripa B., Gargouri N., Willingham A.L., Stein C. (2014). The Global Burden of Foodborne Parasitic Diseases: An Update. Trends Parasitol..

[7] Kaminsky R., Mäser P. (2025). Global Impact of Parasitic Infections and the Importance of Parasite Control. Front. Parasitol..

[8] Van de Vuurst P., Escobar L.E. (2023). Climate Change and Infectious Disease: A Review of Evidence and Research Trends. Infect. Dis. Poverty.

[9] Ge Y., Liu H., Ren N., Qadeer A., Tabios I.K.B., Fontanilla I.K.C., Leonardo L.R., Sripa B., Cheng G. (2025). The Global Prevalence of and Factors Associated with Parasitic Coinfection in People Living with Viruses: A Systematic Review and Meta-Analysis. Pathogens.

[10] Taye B., Desta K., Ejigu S., Dori G.U. (2014). The Magnitude and Risk Factors of Intestinal Parasitic Infection in Relation to Human Immunodeficiency Virus Infection and Immune Status at ALERT Hospital, Addis Ababa, Ethiopia. Parasitol. Int..

[11] Brown M., Mawa P.A., Kaleebu P., Elliott A.M. (2006). Helminths and HIV Infection: Epidemiological Observations on Immunological Hypotheses. Parasite Immunol..

[12] Chulanetra M., Chaicumpa W. (2021). Revisiting the Mechanisms of Immune Evasion Employed by Human Parasites. Front. Cell Infect. Microbiol..

[13] Thakur A., Mikkelsen H., Jungersen G. (2019). Intracellular Pathogens: Host Immunity and Microbial Persistence Strategies. J. Immunol. Res..

[14] Ajibola O., Gulumbe B.H., Eze A.A., Obishakin E. (2018). Tools for Detection of Schistosomiasis in Resource Limited Settings. Med. Sci..

[15] Fasogbon I.V., Ondari E.N., Tusubira D., Ogbonnia E.C., Ashley J., Sasikumar S., Aja P.M. (2024). A Critical Review of the Limitations of Current Diagnostic Techniques for Schistosomiasis. All Life.

[16] Fik-Jaskółka M.A., Mkrtchyan A.F., Saghyan A.S., Palumbo R., Belter A., Hayriyan L.A., Simonyan H., Roviello V., Roviello G.N. (2020). Spectroscopic and SEM evidences for G4-DNA binding by a synthetic alkyne-containing amino acid with anticancer activity. Spectrochim. Acta A Mol. Biomol. Spectrosc..

[17] Roviello V., Musumeci D., Mokhir A., Roviello G.N. (2021). Evidence of protein binding by a nucleopeptide based on a thymine-decorated L-diaminopropanoic acid through CD and in silico studies. Curr. Med. Chem..

[18] Marzano M., Falanga A.P., Marasco D., Borbone N., D’Errico S., Piccialli G., Roviello G.N., Oliviero G. (2020). Evaluation of an Analogue of the Marine ε-PLL Peptide as a Ligand of G-quadruplex DNA Structures. Mar. Drugs.

[19] Roviello G.N., Ricci A., Bucci E.M., Pedone C. (2011). Synthesis, biological evaluation and supramolecular assembly of novel analogues of peptidyl nucleosides. Mol. BioSyst..

[20] Sargsyan A.S., Karapetyan L.T., Mkhitaryan A.V., Stepanyan L.A., Sargsyan T.H., Danghyan Y.M., Sargsyan A.V., Oganezova G.G., Hovhannisyan N.A. (2024). Modeling, Synthesis and In Vitro Testing of Peptides Based on Unusual Amino Acids as Potential Antibacterial Agents. Biomed. Khim..

[21] Roviello G.N., Musumeci D., Bucci E.M., Pedone C. (2011). Evidences for supramolecular organization of nucleopeptides: Synthesis, spectroscopic and biological studies of a novel dithymine L-serine tetrapeptide. Mol. BioSyst..

[22] Roviello G., Musumeci D., Pedone C., Bucci E.M. (2010). Synthesis, characterization and hybridization studies of an alternate nucleo-ε/γ-peptide: Complexes formation with natural nucleic acids. Amino Acids.

[23] Musumeci D., Oliviero G., Roviello G.N., Bucci E.M., Piccialli G. (2012). G-quadruplex-forming oligonucleotide conjugated to magnetic nanoparticles: Synthesis, characterization, and enzymatic stability assays. Bioconjug. Chem..

[24] Roviello G.N., Di Gaetano S., Capasso D., Cesarani A., Bucci E.M., Pedone C. (2010). Synthesis, spectroscopic studies and biological activity of a novel nucleopeptide with Moloney murine leukemia virus reverse transcriptase inhibitory activity. Amino Acids.

[25] Liu L.X., Weller P.F. (1996). Antiparasitic drugs. N. Engl. J. Med..

[26] Malonis R.J., Lai J.R., Vergnolle O. (2020). Peptide-Based Vaccines: Current Progress and Future Challenges. Chem. Rev..

[27] Koger-Pease C., Perera D.J., Ndao M. (2023). Recent Advances in the Development of Adenovirus-Vectored Vaccines for Parasitic Infections. Pharmaceuticals.

[28] Chu K.B., Quan F.S. (2021). Virus-Like Particle Vaccines Against Respiratory Viruses and Protozoan Parasites. Curr. Top. Microbiol. Immunol..

[29] Sukiasyan A., Keshishyan A., Manukyan D., Melik-Andreasyan G., Atshemyan L., Apresyan H., Strelkova M., Frohme M., Cortes S., Kuhls K. (2019). Re-Emerging Foci of Visceral Leishmaniasis in Armenia—First Molecular Diagnosis of Clinical Samples. Parasitology.

[30] Vilas-Boas D.F., Nakasone E.K.N., Gonçalves A.A.M., Lair D.F., Oliveira D.S.d., Pereira D.F.S., Silva G.G., Conrado I.d.S.S., Resende L.A., Zaldívar M.F. (2024). Global Distribution of Canine Visceral Leishmaniasis and the Role of the Dog in the Epidemiology of the Disease. Pathogens.

[31] Kuhls K., Moskalenko O., Sukiasyan A., Manukyan D., Melik-Andreasyan G., Atshemyan L., Apresyan H., Strelkova M., Jaeschke A., Wieland R. (2021). Microsatellite-based molecular epidemiology of *Leishmania infantum* in Armenia reveals re-emerging endemic foci of visceral leishmaniasis in Armenia and pilot risk assessment by ecological niche modeling. PLoS Negl. Trop. Dis..

[32] Paronyan L., Apresyan H., Galstyan A., Keshishyan A., Vanyan A. (2015). Trends in visceral leishmaniasis in Armenia 1999–2014. In International Conference on Emerging Infectious Diseases 2015 poster and oral presen-tation abstacts. Emerg. Inf. Dis..

[33] Aghayan S.A., Grigoryan G., Gevorgyan H., Harutyunyan T., Rukhkyan M., Muradyan V., Karadjian G., Marsot M., Moutailler S., Pollet T. (2024). Diversity and Distribution of Bacterial and Parasitic Tick-Borne Pathogens in Armenia, Transcaucasia. Iran. J. Public Health.

[34] Manucharyan A., Achenbach J., Paronyan L., Avetisyan L., Danielyan R., Melik-Andreasyan G. (2023). Gamasid Ticks as Vectors of Tularemia in the Southeast of Armenia. Vector Borne Zoonotic Dis..

[35] Avetisian L.M. (2004). Sovremennoe sostoianie épidemiologicheskogo nadzora za vazhneĭshimi parazitozami v respublike Armeniia [Epidemiological Surveillance of Parasitic Diseases in the Republic of Armenia]. Med. Parazitol..

[36] Manukyan A., Grigoryan G., Gevorgyan K., Melik-Andreasyan G., Keshishyan A., Sahakyan G. (2025). The Potential Risk Factors of Cystic Echinococcosis in Armenia. Iran. J. Parasitol..

[37] Manukyan A., Avetisyan L., Sahakyan G., Paez Jimenez A., Paronyan L., Gevorgyan K., Vanyan A. (2022). Epidemiological Surveillance of Human Alveolar Echinococcosis in the Republic of Armenia from 2008 to 2020: A Review. Parasite Epidemiol. Control.

[38] Turki H., Shamseddin J., Pournasrolla N., Kahrizi S., Kahrizi S., Kazemi E. (2025). Molecular and cellular aspects of medical parasitology: Insights into pathogenesis and host interactions. Cell. Mol. Biomed. Rep..

[39] Bargieri D., Lagal V., Andenmatten N., Tardieux I., Meissner M., Ménard R. (2014). Host Cell Invasion by Apicomplexan Parasites: The Junction Conundrum. PLoS Pathog..

[40] Silva M.T. (2012). Classical Labeling of Bacterial Pathogens According to Their Lifestyle in the Host: Inconsistencies and Alternatives. Front. Microbiol..

[41] Sojkova P.B., Butenko A., Richtova J., Fiala I., Obornik M., Lukes J. (2024). Inside the Host: Understanding the Evolutionary Trajectories of Intracellular Parasitism. Annu. Rev. Microbiol..

[42] Silva M.T., Silva Pestana N.T. (2013). The In Vivo Extracellular Life of Facultative Intracellular Bacterial Parasites: Role in Pathogenesis. Immunobiology.

[43] Rodriguez A., Suo X., Liu D., Tang Y.W., Sussman M., Liu D., Poxton I., Schwartzman J. (2024). Classification of Medically Important Parasites. Molecular Medical Microbiology.

[44] van Dooren G.G., McConville M.J. (2025). Nutrient Acquisition by Intracellular Parasitic Protists. Cell Host Microbe.

[45] Mead J.R. (2023). Early Immune and Host Cell Responses to *Cryptosporidium* Infection. Front. Parasitol..

[46] Wang Z.X., Jiao W.J., Yang Y., Liu H.-L., Wang H.-L. (2024). Role of Inflammasomes in *Toxoplasma* and *Plasmodium* Infections. Parasites Vectors.

[47] Batista M.F., Nájera C.A., Meneghelli I., Bahia D. (2020). The Parasitic Intracellular Lifestyle of Trypanosomatids: Parasitophorous Vacuole Development and Survival. Front. Cell Dev. Biol..

[48] Epting C.L., Coates B.M., Engman D.M. (2010). Molecular Mechanisms of Host Cell Invasion by *Trypanosoma cruzi*. Exp. Parasitol..

[49] Silva-Moreira A.L., Serravite A.M., Rios-Barros L.V., de Menezes J.P.B., Horta M.F., Castro-Gomes T. (2025). New Insights into the Life Cycle, Host Cell Tropism, and Infection Amplification of *Leishmania* spp. Infect. Immun..

[50] Shadduck J.A., Orenstein J.M. (1993). Comparative Pathology of Microsporidiosis. Arch. Pathol. Lab. Med..

[51] Bonazzi M. (2024). Editorial: Microbiology and Pathogenesis of Chlamydia, Coxiella, and Rickettsia. Front. Cell. Infect. Microbiol..

[52] Gunn A., Pitt S.J. (2012). Parasitology: An Integrated Approach.

[53] Hill D.E., Chirukandoth S., Dubey J.P. (2005). Biology and Epidemiology of *Toxoplasma gondii* in Man and Animals. Anim. Health Res. Rev..

[54] Ramasamy R. (2014). Zoonotic Malaria—Global Overview and Research and Policy Needs. Front. Public Health.

[55] Auld S.K., Tinsley M.C. (2015). The Evolutionary Ecology of Complex Lifecycle Parasites: Linking Phenomena with Mechanisms. Heredity.

[56] Kara R. “Parasitic Disease”. Encyclopedia Britannica, 21 April 2025. https://www.britannica.com/science/parasitic-disease.

[57] Tamarozzi F., Deplazes P., Casulli A. (2020). Reinventing the Wheel of Echinococcus granulosus sensu lato Transmission to Humans. Trends Parasitol..

[58] Theel E.S., Pritt B.S. (2016). Parasites. Microbiol. Spectr..

[59] Walker D.M., Oghumu S., Gupta G., McGwire B.S., Drew M.E., Satoskar A.R. (2014). Mechanisms of Cellular Invasion by Intracellular Parasites. Cell. Mol. Life Sci..

[60] WHO (2024). Fact Sheet: Vector-Borne Diseases. https://www.who.int/news-room/fact-sheets/detail/vector-borne-diseases.

[61] Breedlove B. (2022). Deadly, Dangerous, and Decorative Creatures. Emerg. Infect. Dis..

[62] Chala B., Hamde F. (2021). Emerging and Re-Emerging Vector-Borne Infectious Diseases and the Challenges for Control: A Review. Front. Public Health.

[63] Carbone G., De Bona A., Septelici D., Cipri A., Nobilio A., Esposito S. (2025). Beyond Mosquitoes: A Review of Pediatric Vector-Borne Diseases Excluding Malaria and Arboviral Infections. Pathogens.

[64] Obradović Z., Smjecanin E., Pindzo E., Omerović H., Cibo N. (2022). A literature review on vector borne diseases. Int. J. Med. Rev. Case Rep..

[65] Ouologuem D.T., Dara A., Kone A., Ouattara A., Djimde A.A. (2023). *Plasmodium falciparum* Development from Gametocyte to Oocyst: Insight from Functional Studies. Microorganisms.

[66] Sanyaolu A., Okorie C., Mehraban N., Ayodele O., Tshitenge S.K., Knox R., Mullaj E., Nandzo A., El-Samman A., Nesshwat S. (2016). Epidemiology of Zoonotic Diseases in the United States: A Comprehensive Review. J. Infect. Dis. Epidemiol..

[67] Ruybal-Pesántez S., Sáenz F.E., Deed S.L., Johnson E.K., Larremore D.B., Vera-Arias C.A., Tiedje K.E., Day K.P. (2023). Molecular Epidemiology of Continued *Plasmodium falciparum* Disease Transmission after an Outbreak in Ecuador. Front. Trop. Dis..

[68] Chaturvedi R., Biswas S., Bisht K., Sharma A. (2023). The Threat of Increased Transmission of Non-knowlesi Zoonotic Malaria in Humans: A Systematic Review. Parasitology.

[69] Pinto-Ferreira F., Caldart E.T., Pasquali A.K.S., Mitsuka-Breganó R., Freire R.L., Navarro I.T. (2019). Patterns of Transmission and Sources of Infection in Outbreaks of Human Toxoplasmosis. Emerg. Infect. Dis..

[70] Dubey J.P. (2021). Toxoplasmosis of Animals and Humans.

[71] Shapiro K., Bahia-Oliveira L., Dixon B., Dumètre A., de Wit L.A., VanWormer E., Villena I. (2019). Environmental Transmission of *Toxoplasma gondii*: Oocysts in Water, Soil and Food. Food Waterborne Parasitol..

[72] Razakandrainibe R., Ortega Y., La Carbona S. (2023). Editorial: Cryptosporidium, Giardia, Cyclospora, and Toxoplasma—Insights into Their Transmission. Front. Cell. Infect. Microbiol..

[73] Bouzid M., Hunter P.R., Chalmers R.M., Tyler K.M. (2013). Cryptosporidium Pathogenicity and Virulence. Clin. Microbiol. Rev..

[74] Peng X., Murphy T., Holden N.M. (2008). Evaluation of the Effect of Temperature on the Die-Off Rate for *Cryptosporidium parvum* Oocysts in Water, Soils, and Feces. Appl. Environ. Microbiol..

[75] Kubina S., Costa D., Cazeaux C., Villena I., Favennec L., Razakandrainibe R., La Carbona S. (2023). Persistence and Survival of *Cryptosporidium parvum* Oocysts on Lamb’s Lettuce Leaves during Plant Growth and in Washing Conditions of Minimally-Processed Salads. Int. J. Food Microbiol..

[76] Checkley W., White A.C., Jaganath D., Arrowood M.J., Chalmers R.M., Chen X.-M., Fayer R., Griffiths J.K., Guerrant R.L., Hedstrom L. (2015). A review of the global burden, novel diagnostics, therapeutics, and vaccine targets for cryptosporidium. Lancet Infect. Dis..

[77] McManus D.P., Gray D.J., Zhang W., Yang Y. (2012). Diagnosis, Treatment, and Management of Echinococcosis. BMJ.

[78] Schneider C., Kratzer W., Binzberger A., Schlingeloff P., Baumann S., Romig T., Schmidberger J. (2023). *Echinococcus multilocularis* and other zoonotic helminths in red foxes (*Vulpes vulpes*) from a southern German hotspot for human alveolar echinococcosis. Parasites Vectors.

[79] McCluskey J.M., Sato A.I. (2024). Vertical Transplacental Infections. StatPearls [Internet].

[80] Carlier Y., Truyens C., Deloron P., Peyron F. (2012). Congenital parasitic infections: A review. Acta Trop..

[81] da Silva R.J., Cabo L.F., Boyle J.P. (2024). Teratogenic parasites: Disease mechanisms and emerging study models. Trends Parasitol..

[82] Cevallos A.M., Hernández R. (2014). Chagas’ disease: Pregnancy and congenital transmission. Biomed Res. Int..

[83] Faral-Tello P., Pagotto R., Bollati-Fogolín M., Francia M.E. (2023). Modeling the human placental barrier to understand *Toxoplasma gondii*’s vertical transmission. Front. Cell. Infect. Microbiol..

[84] Chaudhry S.A., Gad N., Koren G. (2014). Toxoplasmosis and pregnancy. Can. Fam. Physician.

[85] Avelino M.M., Amaral W.N., Rodrigues I.M., Rassi A.R., Gomes M.B., Costa T.L., Castro A.M. (2014). Congenital toxoplasmosis and prenatal care state programs. BMC Infect. Dis..

[86] Khairullah A.R., Kurniawan S.C., Widodo A., Effendi M.H., Hasib A., Silaen O.S.M., Ramandinianto S.C., Moses I.B., Riwu K.H.P., Afnani D.A. (2024). A comprehensive review of toxoplasmosis: Serious threat to human health. Open Public Health J..

[87] Bua J., Volta B.J., Velazquez E.B., Ruiz A.M., De Rissio A.M., Cardoni R.L. (2012). Vertical transmission of *Trypanosoma cruzi* infection: Quantification of parasite burden in mothers and their children by parasite DNA amplification. Trans. R. Trop. Med. Hyg..

[88] Herrera Choque A.G., Cuna W.R., Gabrielli S., Mattiucci S., Passera R., Rodriguez C. (2024). Parasitic Effects on the Congenital Transmission of *Trypanosoma cruzi* in Mother–Newborn Pairs. Microorganisms.

[89] Kemmerling U., Osuna A., Schijman A.G., Truyens C. (2019). Congenital transmission of *Trypanosoma cruzi*: A review about the interactions between the parasite, the placenta, the maternal and the fetal/neonatal immune responses. Front. Microbiol..

[90] Cáceres T.M., Patiño L.H., Ramírez J.D. (2025). Understanding Host–Pathogen Interactions in Congenital Chagas Disease Through Transcriptomic Approaches. Pathogens.

[91] Minwuyelet A., Yewhalaw D., Siferih M., Atenafu G. (2025). Current update on malaria in pregnancy: A systematic review. Trop. Dis. Travel Med. Vaccines.

[92] Yeika E.V., Schlemmer B., Abanda M.H., Tolefac P.N. (2017). Congenital *Plasmodium falciparum* malaria: A report of three cases. Clin. Pediatr. Res..

[93] Omidiji M.O., Lesi F.E.A., Esezobor C., Fajolu I.B., Oyibo W.A., Daramola A. (2025). Prevalence of congenital malaria in an urban and a semirural area in Lagos: A two-centre cross-sectional study. Sci. Rep..

[94] Donahoe S.L., Lindsay S.A., Krockenberger M., Phalen D., Šlapeta J. (2015). A review of neosporosis and pathologic findings of *Neospora caninum* infection in wildlife. Int. J. Parasitol. Parasites Wildl..

[95] Rojas-Pirela M., Medina L., Rojas M.V., Liempi A.I., Castillo C., Pérez-Pérez E., Guerrero-Muñoz J., Araneda S., Kemmerling U. (2021). Congenital transmission of apicomplexan parasites: A review. Front. Microbiol..

[96] Elwell C., Mirrashidi K., Engel J. (2016). Chlamydia cell biology and pathogenesis. Nat. Rev. Microbiol..

[97] Robert-Gangneux F. (2012). Dardé M Epidemiology of and Diagnostic Strategies for Toxoplasmosis. Clin. Microbiol. Rev..

[98] Maurin M., Raoult D. (1999). Q Fever. Clin. Microbiol. Rev..

[99] Pexara A., Solomakos N., Govaris A. (2018). Q fever and prevalence of *Coxiella burnetii* in milk. Trends Food Sci. Technol..

[100] Ullah H., Qadeer A., Rashid M., Rashid M.I., Cheng G. (2020). Recent advances in nucleic acid-based methods for detection of helminth infections and the perspective of biosensors for future development. Parasitology.

[101] Baltrušis P., Höglund J. (2023). Digital PCR: Modern solution to parasite diagnostics and population trait genetics. Parasites Vectors.

[102] Jang W.S., Choi M.K., Choe Y.L., Lim C.S. (2025). Development of a Rapid Malaria LAMP-MS Assay for Diagnosis of Malaria Infections. Sci. Rep..

[103] Pomari E., Piubelli C., Perandin F., Bisoffi Z. (2019). Digital PCR: A new technology for diagnosis of parasitic infections. Clin. Microbiol. Infect..

[104] Agut H., Gautheret-Dejean A. (2006). Serologic Testing for Acute and Chronic Infection. Perspect. Med. Virol..

[105] Iatta R., Carbonara M., Morea A., Trerotoli P., Benelli G., Nachum-Biala Y., Mendoza-Roldan J.A., Cavalera M.A., Baneth G., Bandi C. (2023). Assessment of the Diagnostic Performance of Serological Tests in Areas Where *Leishmania infantum* and *Leishmania tarentolae* Occur in Sympatry. Parasites Vectors.

[106] Fitri L.E., Widaningrum T., Endharti A.T., Prabowo M.H., Winaris N., Nugraha R.Y.B. (2022). Malaria Diagnostic Update: From Conventional to Advanced Method. J. Clin. Lab. Anal..

[107] McCaffery J.N., Nace D., Herman C., Singh B., Sompwe E.M., Nkoli P.M., Ngoyi D.M., Kahunu G.M., Halsey E.S., Rogier E. (2021). *Plasmodium falciparum pfhrp2* and *pfhrp3* gene deletions among patients in the DRC enrolled from 2017 to 2018. Sci. Rep..

[108] Krueger T., Ikegbunam M., Lissom A., Sandri T.L., Ntabi J.D.M., Djontu J.C., Baina M.T., Lontchi R.A.L., Maloum M., Ella G.Z. (2023). Low Prevalence of *Plasmodium falciparum* Histidine-Rich Protein 2 and 3 Gene Deletions—A Multiregional Study in Central and West Africa. Pathogens.

[109] Montoya J.G., Liesenfeld O. (2004). Toxoplasmosis. Lancet.

[110] Makarani N., Bharadava K., Kaushik A., Dave A., Gangawane A.K., Kaushal R.S. (2025). Leishmaniasis: A Multifaceted Approach to Diagnosis, Maladies, Drug Repurposing and Way Forward. Microbe.

[111] Robert M.G., Brenier-Pinchart M.P., Garnaud C., Fricker-Hidalgo H., Pelloux H. (2021). Molecular diagnosis of toxoplasmosis: Recent advances and a look to the future. Expert Rev. Anti-Infect. Ther..

[112] Kim M., Park S.J., Park H. (2024). Trend in serological and molecular diagnostic methods for *Toxoplasma gondii* infection. Eur. J. Med. Res..

[113] Switaj K., Master A., Skrzypczak M., Zaborowski P. (2005). Recent trends in molecular diagnostics for *Toxoplasma gondii* infections. Clin. Microbiol. Infect..

[114] Fadel E.F., El-Hady H.A., Ahmed A.M., Tolba M.E.M. (2024). Molecular Diagnosis of Human Toxoplasmosis: The State of the Art. J. Parasit. Dis..

[115] Galluzzi L., Ceccarelli M., Diotallevi A., Menotta M., Magnani M. (2018). Real-time PCR applications for diagnosis of leishmaniasis. Parasites Vectors.

[116] Cardoso L., Mendão C., Madeira de Carvalho L. (2012). Prevalence of *Dirofilaria immitis, Ehrlichia canis, Borrelia burgdorferi* sensu lato, *Anaplasma* spp. and *Leishmania infantum* in apparently healthy and CVBD-suspect dogs in Portugal—A national serological study. Parasites Vectors.

[117] Adams E.R., Schoone G., Versteeg I., Gomez M.A., Diro E., Mori Y., Perlee D., Downing T., Saravia N., Assaye A. (2018). Development and Evaluation of a Novel Loop-Mediated Isothermal Amplification Assay for Diagnosis of Cutaneous and Visceral Leishmaniasis. J. Clin. Microbiol..

[118] Van der Auwera G., Dujardin J.C. (2015). Species Typing in Dermal Leishmaniasis. Clin. Microbiol. Rev..

[119] Hijjawi N., Kanani K.A., Rasheed M., Atoum M., Abdel-Dayem M., Irhimeh M.R. (2016). Molecular Diagnosis and Identification of *Leishmania* Species in Jordan from Saved Dry Samples. Biomed Res. Int..

[120] Liu H., Xie Y., An X., Xu D., Cai S., Chu C., Liu G. (2025). Advances in Novel Diagnostic Techniques for Alveolar Echinococcosis. Diagnostics.

[121] Zhang X., Li Y., Wang H., Chen J., Zhao L., Liu Q. (2024). Circulating Free DNA as a Diagnostic Marker for Echinococcosis: A Systematic Review and Meta-Analysis. Front. Microbiol..

[122] Otero-Abad B., Armua-Fernandez M.T., Deplazes P., Torgerson P.R., Hartnack S. (2017). Latent Class Models for *Echinococcus multilocularis* Diagnosis in Foxes in Switzerland in the Absence of a Gold Standard. Parasites Vectors.

[123] World Health Organization (2017). Global Vector Control Response 2017–2030.

[124] Rahi M., van den Berg H., Vythilingam I., Velayudhan R. (2024). Insect Vectors on the Move. One Earth.

[125] Tourapi C., Tsioutis C. (2022). Circular Policy: A New Approach to Vector and Vector-Borne Diseases’ Management in Line with the Global Vector Control Response (2017–2030). Trop. Med. Infect. Dis..

[126] Ni J., Wang J., Zhang W., Chen E., Gao Y., Sun J., Huang W., Xia J., Zeng W., Guo J. (2025). Sustainable Control and Integrated Management through a One Health Approach to Mitigate Vector-Borne Disease. One Health.

[127] Wilson A.L., Dhiman R.C., Kitron U., Scott T.W., van den Berg H., Lindsay S.W. (2014). Benefit of Insecticide-Treated Nets, Curtains and Screening on Vector-Borne Diseases, Excluding Malaria: A Systematic Review and Meta-Analysis. PLoS Negl. Trop. Dis..

[128] Pryce J., Richardson M., Lengeler C. (2018). Insecticide-Treated Nets for Preventing Malaria. Cochrane Database Syst. Rev..

[129] Lengeler C. (2004). Insecticide-Treated Bed Nets and Curtains for Preventing Malaria. Cochrane Database Syst. Rev..

[130] World Health Organization (WHO) (2021). Malaria Vaccine Implementation Programme.

[131] Ballou W.R., Cahill C.P. (2007). Two Decades of Commitment to Malaria Vaccine Development: GlaxoSmithKline Biologicals. Am. J. Trop. Med. Hyg..

[132] Egbewande O.M. (2022). The RTS, S Malaria Vaccine: Journey from Conception to Recommendation. Public Health Pract..

[133] Vanderberg J.P. (2009). Reflections on Early Malaria Vaccine Studies, the First Successful Human Malaria Vaccination, and Beyond. Vaccine.

[134] Casares S., Brumeanu T.-D., Richie T.L. (2010). The RTS, S Malaria Vaccine. Vaccine.

[135] Sinnis P., Fidock D.A. (2022). The RTS, S Vaccine—A Chance to Regain the Upper Hand against Malaria?. Cell.

[136] World Health Organization (2021). WHO Recommends Groundbreaking Malaria Vaccine for Children at Risk. https://www.who.int/news/item/06-10-2021-who-recommends-groundbreaking-malaria-vaccine-for-children-at-risk.

[137] Feehan J., Plebanski M., Apostolopoulos V. (2025). Recent Perspectives in Clinical Development of Malaria Vaccines. Nat. Commun..

[138] McDaniel J.R., Voss W.N., Bowyer G., Rush S.A., Spencer A.J., Bellamy D., Ulaszewska M., Goike J., Gregory S., King C.R. (2025). Repertoire, function, and structure of serological antibodies induced by the R21/Matrix-M Malaria Vaccine. J. Exp. Med..

[139] Osoro C.B., Ochodo E., Kwambai T.K., Otieno J.A., Were L., Sagam C.K., Owino E.J., Kariuki S., Ter Kuile F.O., Hill J. (2024). Policy Uptake and Implementation of the RTS, S/AS01 Malaria Vaccine in Sub-Saharan African countries: Status Two Years Following the WHO Recommendation. BMJ Glob. Health.

[140] Ouattara A., Laurens M.B. (2015). Vaccines against Malaria. Clin. Infect. Dis..

[141] WHO (World Health Organization) (2023). WHO Recommends R21/Matrix-M Vaccine for Malaria Prevention in Updated Advice on Immunization. https://www.who.int/news/item/02-10-2023-who-recommends-r21-matrix-m-vaccine-for-malaria-prevention-in-updated-advice-on-immunization.

[142] Asante K.P., Mathanga D.P., Milligan P., Akech S., Oduro A., Mwapasa V., Moore K.A., Kwambai T.K., Hamel M.J., Gyan T. (2024). Feasibility, Safety, and Impact of the RTS,S/AS01E Malaria Vaccine When Implemented through National Immunisation Programmes: Evaluation of Cluster-Randomised Introduction of the Vaccine in Ghana, Kenya, and Malawi. Lancet.

[143] Sallam M., Al-Khatib A.O., Al-Mahzoum K.S., Abdelaziz D.H., Sallam M. (2025). Current Developments in Malaria Vaccination: A Concise Review on Implementation, Challenges, and Future Directions. Clin. Pharmacol..

[144] Ilani P., Nyarko P.B., Camara A., Amenga-Etego L.N., Aniweh Y. (2025). PfRH5 Vaccine: From the Bench to the Vial. NPJ Vaccines.

[145] van der Boor S.C., Smit M.J., van Beek S.W., Ramjith J., Teelen K., van de Vegte-Bolmer M., van Gemert G.-J., Pickkers P., Wu Y., Locke E. (2022). Safety, Tolerability, and *Plasmodium falciparum* Transmission-Reducing Activity of Monoclonal Antibody TB31F: A Single-Centre, Open-Label, First-in-Human, Dose-Escalation, Phase 1 Trial in Healthy Malaria-Naive Adults. Lancet Infect. Dis..

[146] Karmakar S., Ismail N., Oliveira F., Oristian J., Zhang W.W., Kaviraj S., Singh K.P., Mondal A., Das S., Pandey K. (2021). Preclinical validation of a live attenuated dermotropic Leishmania vaccine against vector transmitted fatal visceral leishmaniasis. Commun. Biol..

[147] Karmakar S., Volpedo G., Zhang W.W., Lypaczewski P., Ismail N., Oliveira F., Oristian J., Meneses C., Gannavaram S., Kamhawi S. (2022). Centrin-deficient *Leishmania mexicana* confers protection against Old World visceral leishmaniasis. NPJ Vaccines.

[148] Rabienia M., Roudbari Z., Ghanbariasad A., Farjadfar A., Mortazavidehkordi N. (2025). Expression and immunogenicity evaluation of a novel Lentiviral multi-epitope vaccine against Leishmania major in BALB/c mice. Trop. Dis. Travel. Med. Vaccines..

[149] Lim L.Z., Ee S., Fu J., Tan Y., He C.Y., Song J. (2017). Kinetoplastid membrane protein-11 adopts a four-helix bundle fold in DPC micelle. FEBS Lett..

[150] Wang Y., Gao M., Li X., Zhu W., Zhao M., Li J., Liu X., Cao L., Li S., Zhang S. (2023). Structural analyses of a dominant *Cryptosporidium parvum* epitope presented by H-2Kb offer new options to combat cryptosporidiosis. mBio.

[151] Younis B.M., Osman M., Khalil E.A., Santoro F., Furini S., Wiggins R., Keding A., Carraro M., Musa A.E., Abdarahaman M.A. (2021). Safety and Immunogenicity of ChAd63-KH Vaccine in Post-Kala-Azar Dermal Leishmaniasis Patients in Sudan. Mol. Ther..

[152] Volpedo G., Bhattacharya P., Gannavaram S., Pacheco-Fernandez T., Oljuskin T., Dey R., Satoskar A.R., Nakhasi H.L. (2022). The History of Live Attenuated *Centrin* Gene-Deleted *Leishmania* Vaccine Candidates. Pathogens.

[153] Dinc R. (2022). Leishmania Vaccines: The Current Situation with Its Promising Aspect for the Future. Korean J. Parasitol..

[154] Oliveira D.S.d., Zaldívar M.F., Gonçalves A.A.M., Resende L.A., Mariano R.M.d.S., Pereira D.F.S., Conrado I.d.S.S., Costa M.A.F., Lair D.F., Vilas-Boas D.F. (2024). New Approaches to the Prevention of Visceral Leishmaniasis: A Review of Recent Patents of Potential Candidates for a Chimeric Protein Vaccine. Vaccines.

[155] Petitdidier E., Pagniez J., Pissarra J., Holzmuller P., Papierok G., Vincendeau P., Lemesre J.-L., Bras-Gonçalves R. (2019). Peptide-based vaccine successfully induces protective immunity against canine visceral leishmaniasis. NPJ Vaccines.

[156] Regina-Silva S., Feres A.M.L.T., França-Silva J.C., Dias E.S., Michalsky É.M., de Andrade H.M., Coelho E.A.F., Ribeiro G.M., Fernandes A.P., Machado-Coelho G.L.L. (2016). Field Randomized Trial to Evaluate the Efficacy of the Leish-Tec^®^ Vaccine against Canine Visceral Leishmaniasis in an Endemic Area of Brazil. Vaccine.

[157] Fernández Cotrina J., Iniesta V., Monroy I., Baz V., Hugnet C., Marañón F., Fabra M., Gómez-Nieto L.C., Alonso C. (2018). A Large-Scale Field Randomized Trial Demonstrates Safety and Efficacy of the Vaccine LetiFend^®^ against Canine Leishmaniosis. Vaccine.

[158] Toepp A., Larson M., Wilson G., Grinnage-Pulley T., Bennett C., Leal-Lima A., Anderson B., Parrish M., Anderson M., Fowler H. (2018). Randomized, Controlled, Double-Blinded Field Trial to Assess *Leishmania* Vaccine Effectiveness as Immunotherapy for Canine Leishmaniosis. Vaccine.

[159] Velez R., Gállego M. (2020). Commercially approved vaccines for canine leishmaniosis: A review of available data on their safety and efficacy. Trop. Med. Int. Health.

[160] de Souza Testasicca M.C., dos Santos M.S., Marques Machado L., Vieira Serufo A., Doro D., Avelar D., Leonardi Tibúrcio A.M., de Freitas Abrantes C., Machado-Coelho G.L.L., Grimaldi G. (2014). Antibody responses induced by Leish-Tec^®^, an A2-based vaccine for visceral leishmaniasis, in a heterogeneous canine population. Vet. Parasitol..

[161] Kaye P.M., Matlashewski G., Mohan S., Le Rutte E., Mondal D., Khamesipour A., Malvolti S. (2023). Vaccine value profile for leishmaniasis. Vaccine.

[162] Song X., Li Y., Wu H., Qiu H., Sun Y. (2024). T-Cell Epitope-Based Vaccines: A Promising Strategy for Prevention of Infectious Diseases. Vaccines.

[163] Leite J.C., Gonçalves A.A.M., de Oliveira D.S., Resende L.A., Boas D.F.V., Ribeiro H.S., Pereira D.F.S., da Silva A.V., Mariano R.M.d.S., Reis P.C.C. (2023). Transmission-Blocking Vaccines for Canine Visceral Leishmaniasis: New Progress and Yet New Challenges. Vaccines.

[164] Vale D.L., Freitas C.S., Martins V.T., Moreira G.J.L., Machado A.S., Ramos F.F., Pereira I.A.G., Bandeira R.S., de Jesus M.M., Tavares G.S.V. (2023). Efficacy of an Immunotherapy Combining Immunogenic Chimeric Protein Plus Adjuvant and Amphotericin B against Murine Visceral Leishmaniasis. Biology.

[165] Lopes K.F., Freire M.L., Murta S.M.F., Oliveira E. (2024). Efficacy of vaccines based on chimeric or multiepitope antigens for protection against visceral leishmaniasis: A systematic review. PLoS Negl. Trop. Dis..

[166] Boussoffara T., Labidi I., Trimèche M., Chelbi I., Dachraoui K., Msallem N., Abdo Saghir Abbas M., Cherni S., Singh K.P., Kaviraj S. (2025). *LmCen*^−^/^−^-Based Vaccine Is Protective against Canine Visceral Leishmaniasis Following Three Natural Exposures in Tunisia. NPJ Vaccines.

[167] Oliveira F., Rowton E., Aslan H., Gomes R., Castrovinci P.A., Alvarenga P.H., Abdeladhim M., Teixeira C., Meneses C., Kleeman L.T. (2015). A sand fly salivary protein Vaccine shows efficacy against vector-transmitted cutaneous Leishmaniasis in Nonhuman primates. Sci. Transl. Med..

[168] Martiniano de Pádua J.A., Ribeiro D., de Aguilar V.F.F., Melo T.F., Bueno L.L., Grossi de Oliveira A.L., Fujiwara R.T., Keller K.M. (2025). Overview of Commercial Vaccines Against Canine Visceral Leishmaniasis: Current Landscape and Future Directions. Pathogens.

[169] Ayala A., Llanes A., Lleonart R., Restrepo C.M. (2024). Advances in *Leishmania* Vaccines: Current Development and Future Prospects. Pathogens.

[170] You H., Jones M.K., Gordon C.A., Arganda A.E., Cai P., Al-Wassiti H., Pouton C.W., McManus D.P. (2022). The mRNA vaccine technology era and the future control of parasitic infections. Clin. Microbiol. Rev..

[171] Wang C., Fu S., Yu X., Zhou H., Zhang F., Song L., Zhao J., Yang Y., Du J., Luo Q. (2024). *Toxoplasma* WH3 Δrop18 Acts as a Live Attenuated Vaccine against Acute and Chronic Toxoplasmosis. NPJ Vaccines.

[172] Peng G.H., Yuan Z.G., Zhou D.H., He X.H., Liu M.M., Yan C., Yin C.C., He Y., Lin R.Q., Zhu X.Q. (2009). *Toxoplasma gondii* Microneme Protein 6 (MIC6) Is a Potential Vaccine Candidate against Toxoplasmosis in Mice. Vaccine.

[173] Liu Q., Zhang Y., Li S., Zheng B. (2023). A novel *Toxoplasma gondii* TGGT1_316290 mRNA-LNP vaccine elicits protective immune response against toxoplasmosis in mice. Front. Microbiol..

[174] Qi W., Yu Y., Yang C., Wang X.J., Jiang Y., Yu Z. (2024). Nanospheres as the delivery vehicle: Novel application of *Toxoplasma gondii* ribosomal protein S2 in PLGA and chitosan nanospheres against acute toxoplasmosis. Front. Immunol..

[175] Chu K.-B., Quan F.-S. (2021). Advances in *Toxoplasma gondii* Vaccines: Current Strategies and Challenges for Vaccine Development. Vaccines.

[176] Chaudhary N., Weissman D., Whitehead K.A. (2021). mRNA vaccines for infectious diseases: Principles, delivery and clinical translation. Nat. Rev. Drug Discov..

[177] Wang Y., Zhang Z., Luo J., Han X., Wei Y., Wei X. (2021). mRNA vaccine: A potential therapeutic strategy. Mol. Cancer.

[178] Gauci C.G., Jenkins D.J., Lightowlers M.W. (2023). Protection against Cystic Echinococcosis in Sheep Using an *Escherichia coli*—Expressed Recombinant Antigen (EG95) as a Bacterin. Parasitology.

[179] Kouguchi H., Matsumoto J., Katoh Y., Oku Y., Suzuki T., Yagi K. (2007). The Vaccination Potential of EMY162 Antigen against *Echinococcus multilocularis* Infection. Biochem. Biophys. Res. Commun..

[180] Tzipori S., Widmer G. (2008). A Hundred-Year Retrospective on Cryptosporidiosis. Trends Parasitol..

[181] Mead J.R. (2014). Prospects for Immunotherapy and Vaccines against *Cryptosporidium*. Hum. Vaccines Immunother..

[182] MSD Animal Health (2023). Bovilis Cryptium eu Approval Press Release. https://www.msd-animal-health.com/2023/11/29/msd-animal-health-receives-eu-approval-of-bovilis-cryptium/.

[183] (2025). DairyProducer.com. New Vaccine Targets Cryptosporidiosis in Calves. https://www.dairyproducer.com/new-vaccine-targets-cryptosporidiosis-in-calves-offering-early-life-protection/.

[184] Innes E.A., Chalmers R.M., Wells B., Pawlowic M.C. (2020). A One Health Approach to Tackle Cryptosporidiosis. Trends Parasitol..

[185] Fan Y., He Y., Li Y., Yin Z., Shi J., Tian T., Shang K., Shi H., Zhang F., Wen H. (2024). Design of a Novel EmTSP-3 and EmTIP Based Multi-Epitope Vaccine against *Echinococcus multilocularis* Infection. Front. Immunol..

[186] Lundström-Stadelmann B., Rostami A., Frey C.F., Torgerson P.R., Riahi S.M., Bagheri K., Kaethner M., Lachenmayer A., Beldi G., Gasser R.B. (2025). Human Alveolar Echinococcosis—Global, Regional, and National Annual Incidence and Prevalence Rates. Clin. Microbiol. Infect..

[187] RTS,S Clinical Trials Partnership (2015). Efficacy and safety of RTS,S/AS01 malaria vaccine with or without a booster dose in infants and children in Africa: Final results of a phase 3, individually randomised, controlled trial. Lancet.

[188] Chen J., Wang Q., He X., Yang B. (2025). Malaria Vaccines: Current Achievements and Path Forward. Vaccines.

[189] Mordmüller B., Sulyok Z., Sulyok M., Molnar Z., Lalremruata A., Calle C.L., Bayon P.G., Esen M., Gmeiner M., Held J. (2022). A PfSPZ vaccine immunization regimen equally protective against homologous and heterologous controlled human malaria infection. NPJ Vaccines.

[190] Martorell S., Ligda P., Rai S., Alward L., Berish R., Weber A., Isaacson W., Millership J., King V., Pardali D. (2025). Efficacy of a Candidate Vaccine against *Leishmania infantum* on Naturally Exposed Dogs to Sand Flies. Vaccine.

[191] European Medicines Agency CaniLeish. EMA—European Public Assessment Report (Veterinary). https://www.ema.europa.eu/en/medicines/veterinary/EPAR/canileish.

[192] Moreno J. (2019). Assessment of Vaccine-Induced Immunity against Canine Visceral Leishmaniasis. Front. Vet. Sci..

[193] Valadares D.G., Kontowicz E., Tang S., Toepp A., Lima A., Larson M., Grinnage-Pulley T., Scorza B., Pessoa-Pereira D., Oleson J. (2025). LeishTec vaccination disrupts vertical transmission of *Leishmania infantum*. PLoS Negl. Trop. Dis..

[194] Coler R.N., Duthie M.S., Hofmeyer K.A., Guderian J., Jayashankar L., Vergara J., Rolf T., Misquith A., Laurance J.D., Raman V.S. (2015). From Mouse to Man: Safety, Immunogenicity and Efficacy of a Candidate Leishmaniasis Vaccine LEISH-F3+GLA-SE. Clin. Transl. Immunol..

[195] Mo A.X., Pesce J., Hall B.F. (2016). Visceral Leishmaniasis Control and Elimination: Is There a Role for Vaccines in Achieving Regional and Global Goals?. Am. J. Trop. Med. Hyg..

[196] Shital S., Madan E., Selvapandiyan A., Ganguly N.K. (2024). An update on recombinant vaccines against leishmaniasis. Indian J. Med. Res..

[197] Kaur S., Kaur T., Joshi J. (2016). Immunogenicity and protective efficacy of DNA vaccine against visceral leishmaniasis in BALB/c mice. J. Biomed. Res..

[198] Bavarsad Ahmadpour N., Dalimi A., Pirestani M. (2022). Evaluation of a Novel Multi-Epitope Peptide Vaccine Candidate from LACK, LeIF, GP63, SMT Antigens of Leishmania major in BALB/c Mice. Arch. Razi Inst..

[199] Li X., Yuan W., He T., Guo R., Du X., He Y., Li X., El-Ashram S., Al-Olayan E.M., Yang N. (2023). Boosting Mouse Defense against Lethal *Toxoplasma gondii* Infection with Full-Length and Soluble SAG1 Recombinant Protein. Vaccines.

[200] Paronyan L., Babayan L., Vardanyan H., Manucharyan A., Papapostolou K.M., Balaska S., Vontas J., Mavridis K. (2024). Molecular monitoring of insecticide resistance in major disease vectors in Armenia. Parasites Vectors.

[201] Kang H.-J., Quan F.-S. (2025). Efficacy of Heterologous Vaccination Using Virus-Like Particles and Vaccinia Virus Containing MIC8 and AMA1 Proteins of *Toxoplasma gondii*. Vaccines.

[202] Zhu L., Lei Z., Xia X., Zhang Y., Chen Y., Wang B., Li J., Li G., Yang G., Cao G. (2021). Yeast Shells Encapsulating Adjuvant AS04 as an Antigen Delivery System for a Novel Vaccine against Toxoplasma gondii. ACS Appl. Mater. Interfaces.

[203] Fang Y.N., Zhou P., Qi W.Y., Yu Y.L., Wang X.J., Jiang Y.C., Zhang L., Yu Y.L., Wang J.D., Yu Z.Q. (2025). Research on inner membrane complex protein 1: A novel nanovaccines against *Toxoplasma gondii*. BMC Vet. Res..

[204] El Bissati K., Zhou Y., Paulillo S.M., Raman S.K., Karch C.P., Reed S., Estes A., Estes A., Lykins J., Burkhard P. (2020). Engineering and characterization of a novel Self Assembling Protein for Toxoplasma peptide vaccine in HLA-A11:01, HLA-A02:01 and HLA-B*07:02 transgenic mice. Sci. Rep..

[205] Asghari A., Shamsinia S., Nourmohammadi H., Majidiani H., Fatollahzadeh M., Nemati T., Irannejad H., Nouri H.R., Ghasemi E., Shams M. (2021). Development of a chimeric vaccine candidate based on *Toxoplasma gondii* major surface antigen 1 and apicoplast proteins using comprehensive immunoinformatics approaches. Eur. J. Pharm. Sci..

[206] Hosseini S.A., Hashemi S., Siamian D., Asghari A., Fathollahzadeh M., Majidiani H., Shahraki I., Safari M.H. (2025). Discovery of novel vaccine candidates based on the immunogenic epitopes derived from *Toxoplasma* membrane proteins. Clin. Exp. Vaccine Res..

[207] Teh-Poot C.F., Alfaro-Chacón A., Pech-Pisté L.M., Rosado-Vallado M.E., Asojo O.A., Villanueva-Lizama L.E., Dumonteil E., Cruz-Chan J.V. (2025). Immunogenicity of *Trypanosoma cruzi* Multi-Epitope Recombinant Protein as an Antigen Candidate for Chagas Disease Vaccine in Humans. Pathogens.

[208] Aboelsoued D., Abdullah H.H.A.M., Megeed K.N.A., Hassan S.E., Toaleb N.I. (2022). Evaluation of a Vaccine Candidate Isolated from *Cryptosporidium parvum* Oocyst in Mice. Vet. World.

[209] Zhou P., Zhou Z., Huayu M., Wang L., Feng L., Xiao Y., Dai Y., Xin M., Tang F., Li R. (2023). A multi-epitope vaccine GILE against *Echinococcus multilocularis* infection in mice. Front. Immunol..

[210] Aghayan S.A., Asikyan M.V., Shcherbakov O., Ghazaryan A., Hayrapetyan T., Malkhasyan A., Gevorgyan H., Makarikov A., Kornienko S., Daryani A. (2024). *Toxoplasma gondii* in rodents and shrews in Armenia, Transcaucasia. Int. J. Parasitol. Parasites Wildl..

[211] Shcherbakov O.V., Aghayan S.A., Gevorgyan H.S., Abgaryan T.A., Gevorgyan R.H., Jiménez-Meléndez A., Robertson L.J. (2024). Preliminary investigations of parasite contamination of water sources in Armenia. Food Waterborne Parasitol..

[212] Naghashyan N.Z., Grigoryan L.H., Mkrtchyan A.R., Hakobyan A.R. (2018). Cryptosporidiosis of farm animals in the Republic of Armenia. SM J. Infect. Dis..

[213] Manukyan A., Avetisyan L., Sahakyan G., Paez A., Paronyan L., Vanyan A. (2021). Epidemiology of Human Alveolar Echinococcosis in Armenia. Eur. J. Public Health.

[214] Manukyan A. (2023). Young Age of Alveolar Echinococcosis Patient in Armenia: A Case Report. Iran. J. Parasitol..

[215] Strelkova M.V., Ponirovsky E.N., Morozov E.N., Zhirenkina E.N., Razakov S.A., Kovalenko D.A., Schnur L.F., Schönian G. (2015). A Narrative Review of Visceral Leishmaniasis in Armenia, Azerbaijan, Georgia, Kazakhstan, Kyrgyzstan, Tajikistan, Turkmenistan, Uzbekistan, the Crimean Peninsula and Southern Russia. Parasites Vectors.

[216] Rossia A.L., Chuelov S.B., Kondratenko N.V., Tselovalnikova E.V., Tebenkov A.V., Kolyagina S.V., Sokolova N.A., Nepokulchitskaya N.V. (2023). A case of visceral leishmaniasis imported from Armenia. Child. Infect..

[217] (2023). Gevorgyan RG (2023) Epizootological situation on toxoplasmosis among livestock animals in the Tavush region of Armenia. Collection of Scientific Articles adapted from the International Scientific Conference: Theory and Practice of Parasitic Disease Control.

[218] Hovsepyan A., Voskanyan K., Semerjian S., Alexanian Y. (1990). Some results of epidemiological and epizootological investigations of toxoplasmosis in Armenian SSR. Med. Sci. Armen..

[219] Aghayan S.A., Asikyan M., Raković M., Stanković D., Fadeev I.V., Gevorgyan H., Shcherbakov O., Arakelyan M., Aghababyan K., Pagheh A.S. (2024). Molecular Detection of *Toxoplasma gondii* (Chromista: Apicomplexa) in the Blood of Passerines (Aves: Passeriformes) in South-Eastern Armenia. Zoologia.

[220] Janibekyan Z., Amiryan S., Antonyan R. (2002). Prevalence of toxoplasmposis in women of childbearing age in Armenia. Med. Sci. Armen..

[221] Nasir S.M.I., Amarasekara S., Wickremasinghe R., Fernando D., Udagama P. (2020). Prevention of Re-Establishment of Malaria: Historical Perspective and Future Prospects. Malar. J..

[222] Davidyants V., Avetisyan L., Ejov M. (2008). Malaria In Armenia: Achievements And Priorities. New Armen. Med. J..

[223] Davidyants V.A., Kondrashin A.V., Vanyan A.V., Morozova L.F., Turbabina N.A., Stepanova E.V., Maksimova M.S., Morozov E.N. (2019). Role of Malaria Partners in Malaria Elimination in Armenia. Malar. J..

[224] World Health Organization (2017). Weekly Epidemiological Record: Progress towards malaria elimination. Wkly. Epidemiol. Rec..

[225] World Health Organization (2023). World Malaria Report 2023: Tracking Progress and Gaps in the Global Response to Malaria. https://www.who.int/teams/global-malaria-programme/reports/world-malaria-report-2023.

[226] World Health Organization (2024). Countries and Territories Certified Malaria-Free by WHO.

